# Distinct domains of ENHANCER OF PINOID hold information for its polarization required for auxin-mediated cotyledon and flower development in *Arabidopsis*

**DOI:** 10.1371/journal.pgen.1011217

**Published:** 2025-06-23

**Authors:** Michaela S. Matthes, Nicole Yun, Miriam Luichtl, Ulrich Büschges, Birgit S. Fiesselmann, Benjamin Strickland, Marietta S. Lehnardt, Kay Schneitz, Klaus Michel, Ramon A. Torres Ruiz

**Affiliations:** 1 Lehrstuhl für Genetik, Wissenschaftszentrum Weihenstephan, Technische Universitaet Muenchen (TUM), Freising, Germany; 2 Centre for Advanced Light Microscopy (CALM), School of Life Sciences, Technische Universitaet Muenchen (TUM), Freising, Germany; 3 Entwicklungsbiologie der Pflanzen, School of Life Sciences, Technische Universitaet Muenchen (TUM), Freising, Germany; Wake Forest University, UNITED STATES OF AMERICA

## Abstract

The *Arabidopsis* ENHANCER OF PINOID (ENP) protein and the AGC-kinase PINOID (PID) synergistically impact on polarization and function of the auxin transporter PIN-FORMED1 (PIN1) required for plant leaf and flower organ development. ENP offers a PID-independent input for PIN-function since *enp pid* double mutants lead to cotyledon- and flower-less plants in contrast to *pid* single mutants, which develop cotyledons and abnormal albeit fertile flowers. This indicates that ENP, which depicts a similar polar localization as PIN1, is a potential interactor of PINs including PIN1. Here we show that the modular structure of ENP predicted by AlphaFold separates the capability for its own cellular polarization and its function linked to polar PIN1 activity. The part of ENP from aa1 to aa470 is subdivided into three structured domains. They are supportive and/or essential for cellular polarity. In contrast, the C-terminus, which is an intrinsically disordered region (IDR), is completely dispensable for polarity but essential for ENP-mediated PIN1-function. FLIM-FRET shows ENP to be closely associated with the plasma membrane and its IDR to significantly interact with PINs. Moreover, the modification status of two prominent phosphorylation sites in the IDR determines ENPs stability and its capability in supporting PIN1. Our results show ENP to be an element in the assumed PIN-multiprotein complex and explain its impact on PID-independent PIN1 activity.

## Author summary

In plants, directional transport to locally concentrate the plant hormone auxin is fundamental for organ development. In *Arabidopsis thaliana*, PINFORMED1 (PIN1) is a major auxin transporter and the AGC-kinase PINOID (PID) functions as a molecular switch for PIN1s apical vs. basal localization in the cell. The activity of ENHANCER OF PINOID (ENP) is a further independent input supporting PIN1 activity. ENP, like PIN1, is apically localized in epidermal cells of growing organs as a peripheral plasma membrane protein. ENP and four additional proteins (MEL1–4), which are rather engaged in internal tissues, form a family linked to auxin related processes. Artificial expression of ENP in foreign tissues reveals, that it contains modules exclusively required for apical vs. basal localization depending on the tissue where it is present. The same is true for at least one MEL (MEL4). ENPs C-terminus is dispensable for cellular localization but essential for supporting PIN1 function and shows significant interaction with PINs. This domain represents an intrinsic disorganized region with target sites whose modification (phosphorylation) impact on ENPs function. ENP and MELs show significant similarity between each other in all parts except their C-termini, which might reflect specific functions.

## Introduction

The plant hormone auxin works as a concentration-dependent signal molecule controlling various plant developmental processes [1,2]. During embryogenesis local auxin concentrations (auxin maxima) are read out to induce the generation of root vs. cotyledons, the embryonic leaves [3]. They also impact on cotyledon number and shape [4]. During post-embryonic development, auxin controls processes such as the generation of leaf and flower primordia [3]. Auxin maxima are organized by a system of auxin influx and efflux carriers. In the cytosol, the main auxin indol-3-acetic acid (IAA) is a charged, membrane-impermeable molecule making its transport predominantly dependent on efflux carrier proteins [5]. The most important efflux carriers are the closely related plasma-membrane (PM) integral PIN-FORMED proteins (PINs) [3,6]. PINs have been shown to be organized as homodimers, which export auxin via a transport mechanism described as elevator-like, their localization indicating the transport direction [7–9]. Correspondingly, PINs are apically polarized in epidermal cells of (aerial) organ primordia, while they adopt a basal orientation pointing towards the root tip in inner tissues [3]. The basal orientation of PIN1 is controlled by GNOM [10]. The apical polarity of PINs is affected by numerous factors. Among these, the site-specific phosphorylation of PINs by different kinases counteracted by phosphatases [11] is essential although it is debated whether phosphorylation *per se* or a complex temporal/spatial pattern of dynamic de-/phosphorylation determines PIN polarity [1,2,12]. The AGCVIII family kinase PINOID (PID) works as a developmental switch for PIN1 polarity and has a crucial role in shoot development [13]. *Pid* single mutants generate pin-like inflorescences, like *pin* single mutants, but also stems with abnormal but fertile flowers providing seedlings with two or three cotyledons [14,15]. This has been attributed to the observation, that *pid* mutants retain some apically polarized PIN1 in the epidermis of cotyledon primordia [16]. In contrast, double mutants of *PID* and *ENHANCER OF PINOID* (*ENP*) completely lack flowers as well as cotyledons, which correlates with a shift of PIN1 to lateral and basal epidermal cell poles [16]. ENP itself is a classical genetic modifier, as a phenotype of *enp* single mutants is barely detectable. The sepals are slightly fused at their basal end and separated for the rest of their structure [16]. A number of mutants in *pid* background leading to (cotyledon) abnormalities uncovered additional genes involved in these processes. This concerns *pin1* itself and genes required in auxin biosynthesis, the Hippo signalling pathway and endosomal sorting [17–21]. Since ordered growth and development of organs is the readout of correct PIN1 function, together these observations suggest the presence of another rather PID-independent input, which contributes to organogenesis.

ENP, also named MACCHI-BOU4/MAB4 [22] or NAKED PINs in YUCCA1/NPY1 [23] and four additional proteins called MAB4/ENP/NPY-Like (MELs) display similarity in the N-terminal and central domain with the NON-PHOTOTROPIC HYPOCOTYL3 (NPH3) protein while their C-termini exhibit considerable divergence [18,22–24]. The expression pattern of *ENP* (*MAB4; NPY1*) vs. *MELs* is complementary. *ENP* is prominent in its epidermal expression while *MELs* are mainly expressed in internal tissues [18,22]. ENP’s apical and MEL’s mainly basal [24] cellular polarities overlap with the known polarities of PINs. This and the phenotypes of *enp pid* and multiple combined *ENP/MEL* mutants suggested a role in auxin transport including (genetic and/or physical) interaction with PINs. In fact, recently *in vitro* pull-down essays of MEL1 with PIN2 and ENP (MAB4/NPY1) with PIN2 indicated physical interaction. In addition, the interaction of MEL1 with PIN2 was shown by FLIM-FRET [25].

In this study, we have focused on the molecular characterization of ENP and its contribution to PIN1 activity. We have studied the sequence and structure of ENP and related MEL/NPY proteins and established a system to analyse various constructs of ENP and MEL4/NPY4. We show, that ENP’s architecture consists of separated modules required for two different functions: first, the capability of ENP for its own polar localization in the cell; second, the support of PIN1 leading to restoration of the *pid* single mutant phenotype in *enp pid* background (“*enp pid* rescue“). For convenience the former function is termed (ENP) “polarity“ and the latter (PIN-supporting) “functionality“ in the following text. The N-terminal and in particular the central region covers ENP’s capability for apical localization. Although the integrity of these parts is necessary for the overall function of ENP, they alone cannot support PIN1. In contrast ENP’s C-terminus, an intrinsically disordered region (IDR), is dispensable for ENP localization but essential for its function supporting PIN1 at the apical plasma membrane domains in the epidermis. A comparison with MEL4/NPY4, which largely lacks a C-terminal part, shows that its similarity to ENP is sufficient for the same polar capabilities (polarity) but not to replace ENP in its PIN1 supporting function (functionality). Furthermore, our data show that the functional strength of the C-terminus increases depending on the integrity and modification of at least two known phosphorylation targets. FRET analyses using PIN2, the structural and functional homolog of PIN1 in the root epidermis, shows that ENP interacts with the cytosolic loop of PINs. The same technique shows that ENP is closely associated with the PM.

## Results

### The ENPs modular structure displays ordered and intrinsically disordered regions

**Fig 1:**
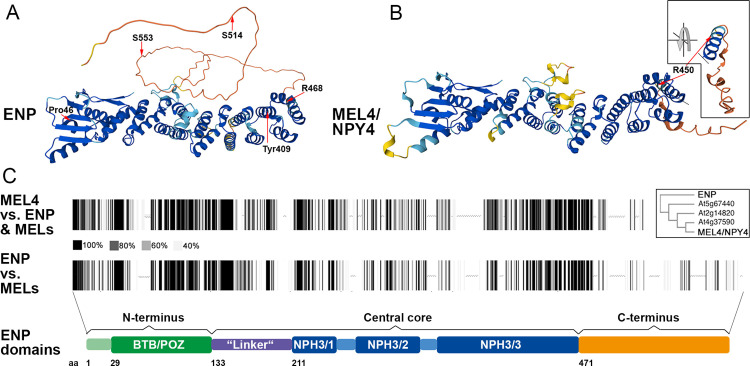
Sequence and structure of ENP and MEL4. A) AlphaFold-predicted structure for ENP [27,28]. B) AlphaFold-predicted structure for MEL4/NPY4. Inset shows turn of the terminal region to highlight position of amino acid R450 (the analogue of R468 in ENP). Studied amino acid residues are indicated with arrows and position. Color code for the per residue confidence metrics in A and B) dark blue: pLDDT>90, light blue: 90 > pLDDT>70, yellow: 70 > pLDDT>50, orange pLDDT<50. C) Protein similarity schemes. The upper bar code compares MEL4 with ENP and the proteins MEL1-3; the lower ENP with MEL1-4; given are gray to black lines, indicating degree of similarity. Note the C-terminal region of ENP vs. the much shorter terminal region of MEL4. The inset depicts a similarity tree between these proteins based on Clustal comparison (https://www.ebi.ac.uk/jdispatcher/msa/clustalo). A schematic drawing indicates the regions tentatively separated by sequence similarity and AlphaFold-predicted structure.

ENP is a protein of 571 amino acids (aas) with a modular architecture (Figs 1, S1 and S2). Multiple protein sequence comparisons with ENP, the MEL/NPY proteins and NPH3 display high, low and no similarity per amino acid residue, which lead to the following differentiation of domains (S1 and S2 Figs). The N-terminus (from aa1 to aa132) contains a BTB/POZ domain (aa29-aa132), which is a conserved protein-protein interaction motif originally found in poxviruses, mice and *Drosophila melanogaster* involved in a variety of functions [26 and references therein]. According to X-ray crystallography data, BTB/POZ domains display tertiary/structural similarity while there is little sequence similarity between different protein families [26]. ENP is a member of plant-specific BTB-NPH3 proteins, whose N-terminus is predicted by AlphaFold to have numerous α -helices and β -sheets with high likelihood as quantified by a residue confidence score called predicted Local Distance Difference Test value (pLDDT) [27,28] (Figs 1A, 1B and S1). These are per residue confidence scores scaled between 0 and 100 indicating how well the predicted structure would agree with the experimental structure. In many BTB-proteins the N-terminal BTB/POZ-domain is followed by a linker region, which connects to the following domain [26]. In ENP, the region from aa133 to aa210 is tentatively designated as “linker“ and the larger region reaching to aa470 as “central core“ with portions of alternating high and low similarity to NPH3 (NPH3_1 to NPH3_3) [26]. The “central core“ contains α-helices of variable length interrupted by only one short region with possible intrinsic disorder (aa185 to aa205). The adjacent C-terminal region (aa 471 to aa571) is quite diverse between ENP and all MELs; in MEL4 it is almost completely missing (Figs 1B, 1C, S1 and S2). By phylogenetic comparison the ENP region from aa1 to aa470, and the corresponding regions from MELs and NPH3 show high similarity while the C-termini are highly dissimilar. This division also exactly overlaps with the structural prediction by AlphaFold [27,28] and a related analysis by AIUPred [29]. AlphaFold indicates intrinsic disorder from aa471 to aa571 by low pLDDTs scores, which have been shown to be suitable predictors for intrinsic disorder of protein regions [30,31]. This is confirmed by AIUPred (S3 Fig), which is a specialized tool for prediction of disordered regions and their binding sites [29]. At the end of ENPs C-terminus a motif of low complexity with numerous serines and arginines (SSSSSSRRRR, aa558-aa567) is conspicuous.

### A genetically based system to efficiently assess polarity vs. functionality of ENP constructs in *enp pid* plants

**Fig 2:**
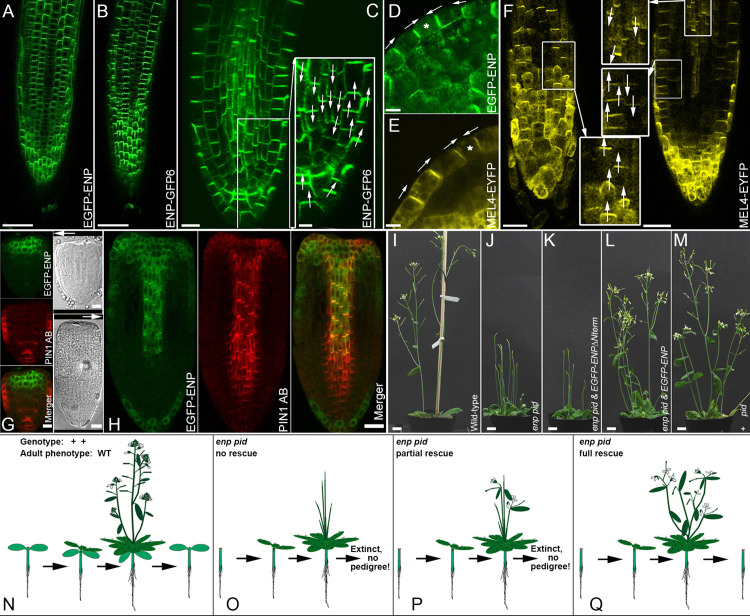
Spatial and temporal expression of ENP and MEL4 constructs and restoration of ENP function. A) EGFP-ENP (focus on epidermis, *35Sp:EGFP-ENP*). B) ENP-GFP6 (focus on epidermis, *35Sp:ENP-GFP6*). C) ENP-GFP6 (focus on internal tissues). D) EGFP-ENP in embryo cotyledon. E) MEL4-EYFP in embryo cotyledon. F) MEL4-EYFP in the seedling root. Left: focus on the epidermis, framed region magnified in the bottom inset; right: focus on the internal tissues/stele, framed regions magnified in the top and middle insets. G) Late *35Sp*-driven EGFP-ENP signal in the central (not lateral) apex region of *enp pid* heart stage embryos (EGFP fluorescence vs. PIN1 AB-Cy3-staining; top: GFP; middle: Cy3; bottom: merger; see S1 Text). H) *35Sp*-driven EGFP-ENP signal proceeds to but does not reach the root tip of *enp pid* torpedo stage embryos. The extension of PIN1 is shown for comparison (Fluorescence/staining as in G). I-M) always *A. thaliana* L*er*-0 ecotype background with I) wild-type plant, J) *enp pid* homozygous plant, K) *enp pid* homozygous plant with non-rescuing construct (*35Sp:EGFP-ENP∆Nterm)*, L) *enp pid* homozygous plant carrying a construct (*35Sp:EGFP-ENP*), which restored ENP function, M) *pid* homozygous *A. thaliana* L*er*-0 ecotype. N) Scheme of full wild-type life cycle. O) Scheme of *enp pid*-plant life cycle with non-functional constructs. The life cycle is the same as that of *enp pid* homozygous plants without any construct, leading to no progeny. P) A life cycle of *enp pid*-plants with constructs competent for partial rescue, i.e., they generate bracts/cauline leaves and flower organs but no seeds leading to extinction. Q) A life cycle of *enp pid*-plants with constructs competent for full rescue thus producing cotyledon-less *enp pid* seedlings, which perpetuate the life cycle. Note, that all plants with constructs cannot generate cotyledons due to late *35Sp* activity in the embryo. Scale bars: 50 µM in A, B; 10 µM in C; 5 µM inset in C, D, E; 20 µM in F, 10 µM in G, 20 µM in H including bottom left, 1 cm in I-M. White arrows indicate polar orientation of EGFP-ENP, ENP-GFP6 and MEL4-EYFP respectively. Star in D and E shows the terminal cell. Here polar ENP and MEL4 respectively, face each other in opposite cells.

For a molecular characterization of ENP, we investigated the impact of its domains on cellular polarity and functionality in terms of rescue of the flower-less phenotype of *enp pid* (formerly designated *laterne* phenotype [16]). The transformation of an *enp pid* double mutant with a fully functional transgenic ENP construct should restore the double mutant to give a *pid* single mutant phenotype. Since the application of pyro-sequencing for the assessment of transgenic and mutant/wild-type genotypes proved to be unsuitable (S4 Fig), we established a genetically based bio-assay, which ensured a *pid enp* double mutant background and simultaneously allowed to assess the cellular localisation and the developmental functionality of constructs. Our approach implements, that ENP is required for both early (embryonic) and late (flower) developmental stages (Fig 2) [16]. In this study, it became essential to use the 35S promoter to drive different constructs for the following reasons. Constructs driven by the 35S promoter are known to be spatially and temporally expressed in an ectopic fashion (Figs 2 and S5). The spatial effect was crucial to assess whether or not ENP localizes basally in internal tissues like its MEL/NPY relatives. The temporal effect was decisive for assuring the *enp pid* homozygous phenotype, as the late onset of the *35Sp*-driven *ENP* expression (S5 Fig) prevents the rescue of cotyledon development. A comparison with PIN1, driven by its endogenous early promoter, illustrates this point in *enp pid* embryos. PIN1 is present in the whole embryo from early on while ENP is lagging behind in the apex (laterally where cotyledon primordia would initiate) and in the root (Fig 2G and 2H). However, the onset of 35S promoter activity in the adult should lead to formation of abnormal but importantly fertile *pid* homozygous flowers in adult development (Fig 2I-M). The different ENP constructs were initially transferred to wild-type plants according to conventional methods (S1 Text). Independent transgenic lines were assessed for ENP-GFP fusion protein presence and signal quality. Selected lines were then further crossed to lines carrying the mutant alleles *enp-1* (*enp*) and *pid-15* (*pid*) [16]. The progeny was selected for lines providing resistant *enp pid* double mutant plants, which were then further used. Thus, the analysis of an ENP construct comprised the following steps. First, selection of antibiotic resistant, cotyledon-less seedlings assured the *enp pid* homozygous background genotype and the presence of the corresponding ENP construct. Second, cellular localization of the GFP-fused protein was assessed by confocal laser scanning microscopy (CLSM) in *enp pid* or (resistant) wild-type siblings. Third, antibiotic-resistant *enp pid* seedlings were grown to maturity. The absence or presence of any organs on stems (bracts, cauline leaves, flower structures) and progeny was scored as no, partial or full functional activity of the introduced ENP version (Fig 2N-Q). The mentioned characteristics in mind, we were aware of the fact that expression of 35S-driven genes deviates from wild-type. We developed this bio-assay to specifically test the capabilities of various ENP variants to partly or fully restore the function of wild-type ENP. The known mutant alleles of ENP are real recessives [16]. The mutant alleles of *PID* and *ENP* used in this study are strong alleles in the *A. thaliana* L*er* ecotype background [16,22]. In the following, we assigned developmental stages of embryos according to Jürgens and Mayer [32].

### ENP displays tissue dependent apical and ectopic basal polarity

ENP and MELs have significant similarity in the N-terminus and central core (Figs 1, S1 and S2). While *ENP* is expressed and apically localized in epidermal cells, *MELs* are mainly expressed in cortex and stele cells where they are basally localized. We analysed whether ENP is capable to polarize in other than epidermal cells. Therefore, “wild-type“, i.e., full-length ENP cDNA-GFP constructs driven by the *35S* promoter were analysed (*35Sp:EGFP-ENP, 35Sp:ENP-GFP6;* Figs 2A-D and S6). The orientation of ENP was apical in epidermal tissue, while it was basal in internal tissues (Fig 2A-D). Expression of ENP itself seemed not to be affected in cells with altered/interrupted auxin transport as given in *enp pid* double mutant embryos. *In situ* hybridization showed epidermal *ENP* mRNA signal as in wild-type (S7 Fig) [22]. Conversely, the overall morphology, especially the development of cotyledons and flowers, of wild-type plants transgenic with *35Sp:EGFP-ENP* or *35Sp:ENP-GFP6* was not disturbed by these constructs (S1 Text and S1 Table and S1 Data). We concluded, that ENP possesses information for polar localization in all cells. The readout of this information differs in epidermal vs. internal tissues leading to apical vs. basal localization, which essentially overlaps with that of PIN1 and PIN2 in accordance with co-immune precipitation results for ENP/MAB4 and PIN2 [25].

### The related MEL4/NPY4 shows the same cellular polarities as ENP

Among the *MEL1–4* (*NPY2–5*) genes, we selected *MEL4/NPY4* (hereafter MEL4) for comparison with *ENP*. Both share some interesting features and also display important differences. Most important is their similarity in the N-terminal and middle regions whereas MEL4 has a very short C-terminus beyond the middle region in comparison to ENP and the other MELs/NPYs (Figs 2E, 2F, S1 and S2). Furthermore, while *ENP* is expressed in the epidermis, *MEL4* is expressed in the stele where the protein displays basal polarity [18,24] (Fig 2A-F). Again, both proteins are internalized to the cytosol upon phenylboronic acid treatment and thus share a similar response to this chemical affecting PM association (S8 Fig) [33,34]. This response is not the same for all PM proteins. For instance, the reaction of HIGH BORON REQUIRING1 (BOR1) was opposite to that of ENP and PIN1 [33].

We tested ectopic expression of *35S*p-driven *MEL4-EYFP* fusion in wild-type torpedo stage embryos and seedling roots. *MEL4* constructs showed weaker fluorescence in comparison to *ENP* constructs but the protein clearly adopted a basal orientation in inner tissues and an apical localisation in the epidermis (Fig 2E and 2F).

Thus, MEL4 also possesses sufficient information for apical and basal polarity in epidermal and inner tissues respectively. The information for apical vs. basal PM localization of ENP and MEL4 (and the other MELs/NPYs) is likely encoded in their N-terminal and/or central core.

### Full length *ENP* constructs restore ENP function in *enp pid* double mutants

**Fig 3:**
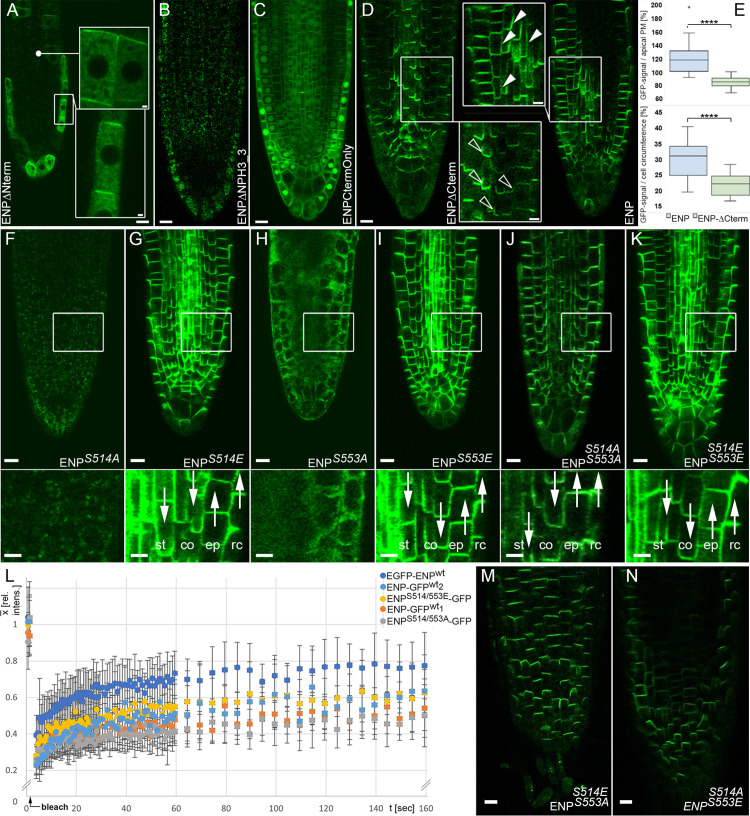
Properties of deletion and point mutation ENP constructs. A-D) Deletion constructs. A) *35Sp:EGFP-ENP∆Nterm construct*. Insets: magnifications. Note, the inset at the top is a magnification of the same specimen in the indicated region at slightly different focus. The shown signals were weak and only few cells displayed their polarity. B) *35Sp:ENP∆NPH3_3-GFP6* construct. C) *35Sp:ENPCtermOnly-GFP6* construct. D) *35Sp:ENP∆Cterm-GFP6* (left) in comparison with full length *35Sp:ENP-GFP6* (right). Insets: open vs. filled arrowheads point to the different lateral extensions (“smile“) of ENP-∆Cterm vs. ENP full length at the PM respectively (cortical and stele cells compared). E) *t*-Test for (lateral) extension of ENP-∆Cterm vs. ENP at the PM (p < 0.0001; see S1 Text). F-K) ENP-GFP localization of point mutations in critical C-terminal sites S514 and S553. Point mutations in the constructs are indicated. White framed regions magnified at the bottom of each image. Auxin flux (as derived by polarity of ENP-GFP) indicated by white arrows in stele (st), cortex (co), epidermis (ep) and root cap (rc) cells. All constructs with *35Sp* and *GFP6*. Brightness and contrast in F and H were strongly elevated to visualize residual GFP signal or make the root visible. L) Mobility of ENP S514/S553 double mutants compared to wild-type ENP as analysed by FRAP. Comparison of wild-type and mutant ENP transformants (indicated). Data generated from FRAP-analysis; at least three independent experiments each. M, N) Polar localization of ENPS514E/S553A (M) and ENPS514A/S553E (N). Scale bars A-D, F-K, M, N: 10 µM, insets in A: 1 µM, in D, F-K: 5 µM.

**Fig 4:**
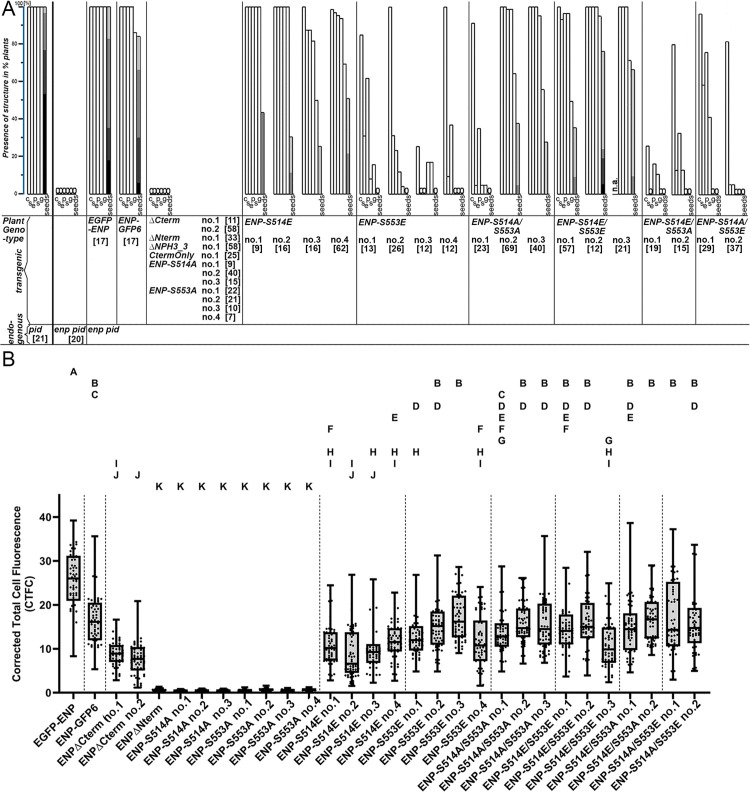
Restoration of ENP function and signal quantification of ENP constructs. A) Frequencies of organs generated and progeny produced by independently transformed lines in homozygous *enp pid* plants (constructs indicated; see main text and S6 Fig). The data for representative *enp pid* double and *pid* single homozygous mutants without constructs are given (many more have been inspected along the course of this study with the same outcome). Number of assessed plants per independent transformed line are given in brackets. Abbreviations are for C: cauline leaves or bracts, S_e_: sepals, P: petals, S: stamens, G. gynoecia, and seeds. Seed production is scored as plants with 1-25 (light grey), 26-100 (medium grey), 101-200 (darker grey) and > 200 (dark grey) seeds. B) Given is the Corrected Total Cell Fluorescence as estimated from at least ten different seedlings (six plasma membranes each) for each independent transformed construct. The boxplots indicate single data points, the median, the minimum, the first quartile, the third quartile and the maximum. The signal intensity was corrected for background for each measurement. One-way Analysis of variance-Tests, followed by Tukey-test for multiple comparison corrections, were performed. The different letters indicate statistically significantly differences (p < 0.05). The different data groups were separated into eleven statistical groups designated A-K, each group representing a distinct subset of data. Boxplots belonging to one statistical group are significantly different to groups belonging to other statistical groups. Boxplots belonging to multiple statistical groups are not significantly different to any of them although they have significant differences to each other.

The full length ENP constructs *35Sp:EGFP-ENP* and *35Sp:ENP-GFP6* were initially transformed into wild-type *A. thaliana* plants. The (not sequence-altered) full length constructs displayed perfect apical vs. basal polarity of ENP in epidermal vs. inner tissues respectively (Fig 2). This was also achieved by some phosphomimic constructs (Fig 3, see below). However, cotyledon-less seedlings carrying EGFP/GFP6 fused to either the N- or C-terminus of ENP displayed the best performance in terms of full restoration of the *enp* mutation (Fig 4A and S2 Data). The adult plants often resembled *pid* single mutants (Fig 2I-Q), sometimes also with respect to seed production. They developed rosette leaves, stems, leaf-like structures and *pid*-like flowers. Notably, they produced 100% cotyledon-less progeny, thus representing a new cotyledon-less plant population (Fig 2Q). The presence of cotyledon-less progeny was already seen by the morphology of the seed (S9 Fig).

### ENPs central core region is required for polar localisation

Next, we tested the significance of the N-terminal region up to the end of the central core for cellular polarity. First, we deleted the N-terminus from aa1 to aa53, which also deletes 25 aas of the BTB/POZ domain (ENP-∆Nterm construct; S1, S2 and S6 Figs). These plants displayed predominantly cytosolic distribution of the GFP signal. However, careful inspection revealed residual GFP signal with apical polarity in few cells of the epidermal layer (Fig 3A). This construct did not restore ENP function (Fig 4A and S2 Data).

We then introduced deletions starting from the C-terminus. One deletion covered the region from aa369 to aa571 (ENP-ΔNPH3_3; S1, S2 and S6 Figs). This deletion resulted in irregularly-distributed cytosolic signal, sometimes in patches, no localisation at the PM (Fig 3B) and no *enp* rescue (Fig 4A and S2 Data).

We analysed two additional constructs. The ENP-CtermOnly construct represented the complete C-terminal intrinsic disordered region, which consist of 100 aas in length (S1, S2 and S6 Figs). It produced an abnormal pattern with strong GFP-localisation in the nucleus and the PM in general and less localisation in the cytosol (Fig 3C). No rescue of *enp* could be observed (Fig 4A and S2 Data).

The ENP-ΔCterm construct spanned aa1 to aa470, the domains for which AlphaFold predicts structure. This ENP-ΔCterm deletion mimicked the original *enp* allele (*enp-1*) [16,22], which converts aa468 (R) into a STOP codon (Figs 1A, S1 and S2). R468 lies at the end of the last alpha-helix predicted by AlphaFold and the last region of similarity between ENP and all MELs (Figs 1A-C, S1 and S2). A homologous residue is also found in MEL4 at position 450, with similar AlphaFold confidence metrics (Figs 1B, S1 and S2). The next amino acids up to aa571 have only very low similarity to MELs. MEL4 almost lacks this part completely. Nevertheless, ENP-ΔCterm plants showed the same cellular polarity pattern as the full length ENP with considerable signal strength (Fig 3D). However, detailed inspection showed that the distribution of this construct was somewhat restricted. Quantitative analysis of GFP-fluorescence of full length ENP vs. ENP-ΔCterm constructs showed that the former displayed stronger extension (“smile“) to lateral sites (Fig 3D, 3E, S3 Data and S10 Fig). Notably, the analysed plants did not show any rescue (Fig 4A and S2 Data). Therefore, we conclude that ENPs N-terminus and especially the central core contains sufficient information for polar localisation. ENP is rarely capable to retain polarity when a considerable part of the N-terminus is deleted (Fig 3).

### Phosphomimetics support Ser^514^ and Ser^553^ in the C-terminal IDR to be critical for ENPs functional capability

The deletion constructs tested showed that the N-terminus and even more the central core of ENP is required for polarity while the C-terminus (aa471-aa571) *per se* is not. Conversely, the N-terminus and central core alone are not capable to restore ENP function, for this the C-terminus is essential.

Besides intrinsic disorder, ENPs C-terminus thus displayed another hallmark of IDRs: that is functionality [35]. Moreover, IDRs have been reported to frequently harbor (functional) phosphorylation sites, especially at serines and threonines [36–38]. For ENP, the phospho-proteome database (PhosPhAt4.0 database; https://phosphat.uni-hohenheim.de/) lists serine target sites, the two most prominent localized in the C-terminus. These were especially conspicuous in three aspects: data quality, abundance and detection in at least three independent studies. The first phosphorylation site [(pS)GGGAQLMPSR] was localized at S514 [39,40]. The second [SSEVSSGSSQ(pS)PPAK] was localized at S553 [39–41]. Both were confirmed in a recent mass spectrometry study with high confidential values [42].

By means of site directed mutagenesis, either phospho-dead exchanges to alanine or phosphomimic exchanges to glutamic acid were introduced giving four different ENP constructs with the exchanges S514A, S514E, S553A and S553E respectively (Fig 3F-I). We evaluated the independent transformant lines separately (Fig 4A and S2 Data) to obtain best information on the impacts of the mutant variants separated from possible transformation/position effects.

Assessment of GFP-signal localization in the progeny revealed (weak) cytosolic distribution without any polar localization of GFP in both S to A single mutant constructs (ENP^S514A^; ENP^S553A^; Fig 3F and 3H), whereas S to E exchanges (ENP^S514E^; ENP^S553E^; Fig 3G and 3I) displayed perfect basal (inner tissues) vs. apical (epidermis) localization of the GFP signal. In none of the lines did single S to A exchanges lead to restoration of ENP function (Fig 4A and S2 Data). In contrast, changes from S to E always led at least to partial rescue, ENP^S514E^ performing significantly better than ENP^S553E^ in terms of flower organ development and seed production (Fig 4A and S2 Data). Few ENP^S514E^ plants could produce (cotyledon-less) progeny in quantities comparable to “wild-type“ *EGFP-ENP* or *ENP-GFP6* constructs (Fig 4A and S2 Data). ENP^S553E^ constructs could generate all flower structures but no progeny (Fig 4A and S2 Data).

With these results in mind, we generated two additional constructs where both serines (at aa514 and aa553) were either replaced by alanines (ENP^S514A/S553A^) or by glutamic acids (ENP^S514E/S553E^). Interestingly, both variants resulted in perfect polarity of ENP-GFP (Fig 3J and 3K). All showed at least partial rescue (including flower organs) and 5/6 lines included plants, which produced cotyledon-less progeny (Fig 4A and S2 Data). Thus, both variants ENP^S514E/S553E^ and ENP^S514A/S553A^ have the capability for complete restoration of ENP function.

On the molecular level, full-length ENP (assumed to be phosphorylated at both sites ENP^S514-P/S553-P^), ENP^S514A/S553A^ (without charge) and ENP^S514E/S553E^ (with charge) should display similar characteristics such as structure, folding, association and mobility at the PM. We addressed this latter aspect with Fluorescence Recovery After Photobleaching (FRAP). Both double phospho-mimetic versions essentially displayed similar recovery dynamics as independent ENP wild-type transformants except a slightly higher recovery for the N-terminal GFP fusion (Fig 3L and S4 Data). Essentially, this pattern remained stable when we altered the diameter of the region to be bleached (S11 Fig and S5 and S6 Data). Note, that diffusion constants and recovery times for FRAP change extremely slow with mass [43], suggesting that the behavior of the GFP-fusion construct approximates that of ENP alone.

In the next step, we tested whether a simple charge imbalance in positions S514 vs. S553 could be a cause for protein instability seen in ENP^S514A^ and ENP^S553A^, which both leave the second site free for phosphorylation. Such a situation is mimicked in the versions ENP^S514A/S553E^ and ENP^S514E/S553A^. However, these variants displayed perfect cellular polarization of ENP (Fig 3M and 3N). They were capable to partially restore ENP function and developed sepals, petals and stamens but not gynoecia and progeny (Fig 4A and S2 Data).

### Quantification of GFP signals of fusion proteins points to the significance of sequence, structure and modification/phosphorylation for ENP function

We subjected all above mentioned constructs to protein quantification at plasma membranes via measurement of the GFP signal strength (Fig 4B and S7 Data). These data have to be considered in context of the capabilities of constructs to restore ENP function (Fig 4A and S2 Data). The unmodified full-length constructs had high signal strength in terms of corrected total cell fluorescence (CTFC) (for details see figure legend, main text and S1 Text). However, the CTFC for ENP-GFP6 for instance covers well with that of some constructs with modifications in S514 and S553 or it is even surpassed (Fig 4B and S7 Data). For instance, this can be seen for constructs ENP-S553E (3^rd^ independent transformant), ENP-S514A/S553A (3^rd^ independent transformant), ENP-S514E/S553E (2^nd^ independent transformant), ENP-S514A/S553E (1^st^ independent transformant) and others (Fig 4B and S7 Data). Other constructs are close to the CTFC values of *35Sp:ENP-GFP6.* Most of these plants perform significantly worse than the unaltered full-length construct (Fig 4A and S2 Data). Although there are exceptions (e.g., ENP∆Nterm), we conclude, that several specific sequence alterations rather than the amount of protein, given by the GFP signals, impact on the strength and capability to restore ENP function of the corresponding constructs.

### Mutation of conserved amino acid residues often retains cellular polarity but impacts severely on functionality

The foregoing analyses demonstrated the significance of the N-terminus/central core for polarity and that of the C-terminus for function. Considering the (non-rescue) effect of the ∆N-term construct for function, we extended the analysis of the former using point mutations of highly conserved amino acids localized in the region aa1 to aa470.

**Fig 5:**
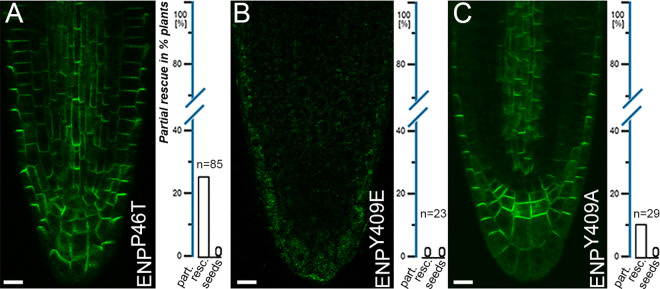
ENP constructs with point mutations in the BTB/POZ and central core region. A-C) Selected plants harboring constructs with point mutations in the N-terminal, linker and central core region. A) *35Sp:ENP*^*P46T*^*-GFP6* construct. B) *35Sp:ENP*^*Y409E*^*-GFP6* construct. C) *35Sp:ENP*^*Y409A*^*-GFP6* construct. Plants with *35Sp:ENP*^*P46T*^*-GFP6* (A) and *35Sp:ENP*^*Y409A*^*-GFP6* construct (C) were capable of partial rescue. The former could generate bracts/cauline leaves and flower structures; the latter only bracts/cauline leaves. Absence of *enp* rescue capability and percentages of partial rescue respectively are indicated in the scale to the right. A-C: 10 µM.

The proline at position 46 in the BTB/POZ domain is a conserved residue in one of two short helical regions, which form a structural turn setting a group of beta-sheets in to a (anti)parallel arrangement (Figs 1A and S1). In some non-plant BTB/POZ proteins, it is a contact site for protein interactions [26] (S1 Fig). Due to its spatial topology, proline confers a characteristic kink in the protein structure. Thus, any replacement of proline should significantly alter the local protein microstructure, which in this case would be the first alpha-helix in ENP (Figs 1A and S1; from aaK44 to aaL48) according to previous structural data [26] and AlphaFold [27]. Plants carrying a threonine in this position (ENP^P46T^) retained perfect cellular polarity (Fig 5A and S2 Data). However, only in 25% of all plants this construct led to partial reversion of the *enp pid* to the *pid* phenotype with bracts/cauline leaves and occasional flower structures (Fig 5A and S2 Data).

We then focused on the well-conserved aa tyrosine 409, which is part of a longer alpha-helix within a group of more or less similarly oriented helices before the start of the IDR (Figs 1A and S1). It is also part of an in-frame GLY deletion mutant (aas407–409) of the (*enp*) *mab4–1* null allele [22]. We considered both potential phosphomimic and phospho-dead versions. The alteration Y409E (ENP^Y409E^) resulted in absence of any localisation, low abundance in the cytosol and no restoration of ENP function (Fig 5B and S2 Data). In contrast, the alteration of Y409A (ENP^Y409A^) retained perfect polarity and achieved 10% partial ENP rescue (i.e., only bracts/cauline leaves formed; Fig 5C and S2 Data). Apparently, the replacement of highly conserved amino acids significantly disturbs the structural integrity of these regions, which is also a requirement for functionality. However, if correctly structured, these regions cannot fulfil ENPs function. This is controlled by the C-terminus.

### Neither MEL4 nor MEL4/ENP-C-terminus domain swaps can restore ENP function

The aforementioned results suggested that the integrity of the region from aa1 to aa470 also contributed to ENP function. We extended these analyses by testing whether MEL4 could functionally replace ENP. MEL4 displays high similarity, with ENP along its stretch from aa1 to aa470 (Figs 1C, S1 and S2). None of the *enp pid* plants carrying *35Sp:MEL4-EYFP* (n = 63) produced any leaf-like structures on stems (S2 Data).

**Fig 6:**
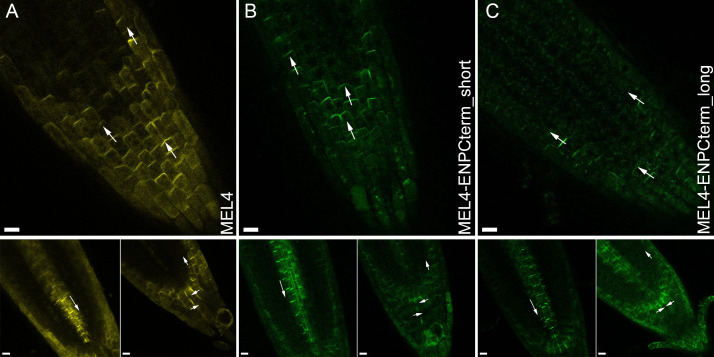
MEL4 and MEL4 with short and long ENP-Cterminal parts. A) CLSM of construct MEL4-EYFP, B) CLSM of construct MEL4-ENPCterm_short-GFP6. C) CLSM of construct MEL4-ENPCterm_long-GFP6*.* The focusses in the subfigure are always the following. Top: focus on epidermis surface, Bottom left: focus on the stele. Bottom right: focus on the root tip, inner cells. Note: orientation of fusion proteins exemplified by arrows in some cells is apical in top figures, basal in bottom left figures and lateral and apical respectively in bottom right figures. Due to the variable expression strength in MEL4 constructs, the figures represent different individuals. Scale bars: 10 µM.

Since MEL4, in comparison to ENP and the other MELs/NPYs, has only a very short C -terminus, we tested whether the addition of the ENP C-terminus could convert MEL4 to a (partially) ENP-functional version. We generated two constructs (Figs 6 and S6 and S1 Text). One construct fused the ENP C-terminus from aa471 to aa571 to the MEL4 protein fragment aa1 to aa452 (*35Sp:MEL4-ENPC term_long-GFP6*). The other was the addition of a slightly shorter ENP C-terminal part (aa500 to aa571) to compensate for the fusion of the complete MEL4 protein (aa1 to aa481) (*35Sp:MEL4-ENPC term_short-GFP6*). All variants were capable to reproduce the apical vs. basal polarity in the epidermis and inner cells respectively (Fig 6). However, like the wild-type MEL4/NPY4, the domain swap variants did not produce any leaf organs above the rosette (n = 14 and n = 34 respectively; S2 Data). Thus, MEL4 is not sufficiently similar to ENP in order to rescue the *enp* mutation even when extended with C-terminal parts of ENP.

### ENP is closely associated with the PM

**Fig 7:**
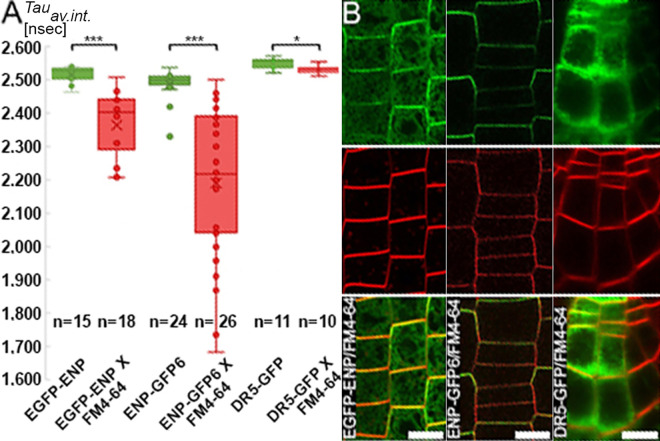
FLIM-FRET analyses of ENP-GFP with FM4-64 as acceptor. A, B) ENP constructs with/without FM4-64 (as indicated). A) ENP with N-terminally and C-terminally fused GFP display significant FRET in presence of FM4-64. In contrast, the cytosolic GFP protein whose expression is driven by the synthetic promoter DR5 shows negligible if any FRET (the range is < 20 psec). B) Representative images showing GFP and FM4-64 fluorescence (separated top and middle) and the merger (bottom).

CLSM shows significant amount of ENP protein close to the PM (Fig 7 and S8 Data). However, with best objectives the maximum resolution in the xy-dimension is approx. 200nm (400nm in z-dimension), which leaves significant space for a distant localization of ENP to the PM. Analysis with various algorithms (ARAMEMNON: http://aramemnon.uni-koeln.de/) does not show any prenylation or related motifs nor trans-membrane domains. However, on the basic hydrophobic (BH) scale [44] ENP displays small potential contact sites along its complete length including the C-terminus (S12 Fig). To experimentally assess potential contact between ENP and the PM we used FLIM-FRET and short (2–5 min) treatments of plants with the PM-dye FM4–64 (as acceptor) and GFPs from *35Sp:EGFP-ENP* and *35Sp:ENP-GFP6* respectively (as donors; Fig 7A, 7B and S8 Data). The obtained lifetime values indicated close association (< 10nm) to the PM for both. Their spread towards low lifetime values (ca. 2.0 nsec) in some specimen indicated fast permeation of FM4–64 into the PM. Lifetime values, with FM4–64 as acceptor, expressed as *τ*_average intensity_ were ca. 2.36 nsec (EGFP-ENP) and ca. 2.19 nsec (ENP-GFP6) as compared to controls without FM4–64, which were ca. 2.52 nsec (EGFP-ENP) and ca. 2.49 nsec (ENP-GFP6; Fig 7A and S8 Data). A control experiment with the cytosolic GFP driven by the synthetic promotor DR5 [3] shows the lifetime values of this GFP in absence and presence of FM4–64 (Fig 7A and S8 Data). The confocal image shows that, in contrast to the first experiments, both fluorophores do not appreciably overlap in the cells (Fig 7B).

### ENP interacts with PIN2 mainly with its C-terminus

Next, a possible FRET with the PM-integral PIN2 auxin efflux carrier was tested. PIN2 instead of PIN1 was chosen for several reasons. First, PIN1 is basally localized in the stele but as such covered by several tissue layers which aggravates the FRET analyses. In contrast, PIN2 is apically localized in epidermal and basally localized in cortex cells. PIN2 is also structurally and functionally related to PIN1, which can even replace PIN2 [45]. Additionally, PIN2 has been shown to co-precipitate with ENP/MAB4 [25].

**Fig 8:**
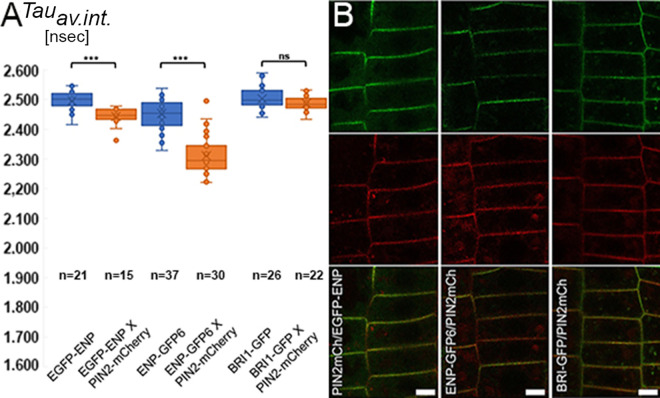
FLIM-FRET analyses of ENP-GFP and PIN2-mCherry as acceptor. A, B) ENP and BRI constructs in absence/presence of PIN2-mCherry (as indicated). A) The *TAU*_*average intensity*_ values of the donor only and donor with acceptor combinations as indicated are given. Differences are significant (p < 0.0001) or not significant (p > 0.05) with one-tailed *t*-tes*t* (see S1 Text). B) representative fluorescence images of EGFP-ENP X PIN2 (left), ENP-GFP6 X PIN2 (middle) and BRI-GFP X PIN2 (right) seedlings showing GFP fluorescence (top), mCherry fluorescence (middle) and the merger (bottom). Scale bars in B: 5 µM.

We performed FLIM-FRET analyses with EGFP-ENP and ENP-GFP6 in combination with a PIN2-mCherry construct [46]. The latter was also combined with BRASSINOSTEROID INSENSITIVE1 (BRI1) (Fig 8A, 8B and S9 Data). The rationale was to show that GFP and mCherry molecules do not necessarily exhibit FRET even when they localize in the same (PM) region such that their fluorescence colours merge in CLSM images.

The *τ*_average intensity_ life time for ENP-GFP6 alone in this experiment was ca. 2.47 nsec while it was ca. 2.33 nsec in presence of PIN2-mCherry (Fig 8A and S9 Data), which gives a difference of 140 psec and an energy transfer rate of E = 5.7%. This is well within the range reported for other cases [25,47–49], considering the Förster distance of R_0_ = 5.288 nm for the (E)GFP-mCherry pair, a distance of approx. 8.4 nm for the GFP at the ENP-C-terminus and the mCherry in the cytosolic loop of PIN2 results (this calculation assumes a *kappa*^*2*^ orientation factor of 2/3 see S1 Text). The measurement of ENP-N-terminus vs. PIN2 exhibits only a difference of 52 psec (E = 2.1%), which is a very weak, borderline FRET (Fig 8A and S9 Data).

Cross-talk is generally known to occur between different (hormonal) signal-transduction pathways. However, cross-talk requires critical fine-tuning. Therefore, we expected absence of interaction between members of auxin signal transduction such as PIN2 and the PM localized brassinosteroid receptor BRI1 [50]. Indeed, FRET between BRI1 and PIN2 was negligible if not absent (Fig 8B and S9 Data). The lifetime difference of BRI1-GFP alone vs. BRI1-GFP combined with PIN2-mCherry was 2.52 nsec vs. 2.50 nsec. Note, that in the CLSM figures the merger of BRI1-GFP and PIN2-mCherry is as visible as for the (EGFP/GFP6-)ENP and PIN2-mCherry fluorophore pairs (Fig 8B). These results strongly suggest that the C-terminus of ENP interacts with PIN2 while the N-terminus is more distantly neighboured.

## Discussion

ENP and MELs play an important role in auxin transport by co-operating indirectly or directly with AGC kinases, in particular PID, and PIN proteins [16,18,22–25]. Considering the number of aforementioned factors, which impact on the developmental effects of auxin linked to the activity of PINs, the list of (in-)direct co-operators of ENP and MELs might extend in the near future. The dissimilarity of their C-termini also suggests a corresponding number of specificities and tasks. Recently, an unexpected observation, described as haplo-complementation, fosters the view of PIN1 being part of a larger protein complex sensitive to PIN1 dosage [51]. Together with all accumulated observations this supports the existence of a PID-independent input or pathway in organogenesis with ENP as an important element.

### ENP likely contacts the PM with different parts

According to the current knowledge, ENPs N-terminus and central core adopt an elongated cylindrical sphere [26–28]. ENP has no obvious lipid modification signals or transmembrane domains. At least, at its termini and its centre GFP integrations analysed in this study would have disturbed such signals. According to scanning searches with the modified EMBOSS program [44] potential contact sites on a BH scale are found along the entire structure of ENP. This might explain why ENP retains significant cellular polarity despite severe deletions and point mutations. For the polarly localized D6 and related protein kinases such contacts (with BH > 0.6 for D6) have been shown in a polybasic lysine-rich motif which binds polyacidic phospholipids of the PM [52].

We analysed membrane association of ENP *in vivo* using FLIM-FRET. FM4–64 is a lipophilic PM stain, which initially localizes at the outer PM leaflet and is useful for studies of endocytosis [53,54]. FM4–64 causes transient internalization of GFP-tagged PM proteins in plant cell culture cells after 10 min treatments but not in the *Arabidopsis thaliana* root [54]. The treatments applied in this study indicate a FRET of ENP-GFP with FM4–64 in the outer leaflet of the PM. The internalisation of FM4–64 by endocytic processes [53,54] cannot be fully excluded but should be marginal given the short (2–5 min) treatments. Considering the dimensions of plant PMs of approximately 6nm (hydrocarbon core and interfacial regions) [55], this is within the distance of FRET (< 10nm) [56]. A possible activity of flippases [57] would transfer FM4–64 to the inner leaflet, thus bringing the dye nearer to ENP. However, FM4–64 does not appreciably flip in the PM and diffusion of FM4–64 into the cytosol could also be excluded [53]. Together, the presented FRET results show ENP being closely neighboured to the PM. The FRET values of ENP-GFPs contrast well with that of the cytosolic GFP protein.

### ENPs information for tissue specific apical vs. basal cellular polarity is buried in the N-terminus and the central core

So far, it was not known whether (and if, where) ENP harboured an inherent determinant for polarity and its recognition by the cellular machinery. Our work shows, that this information is to a large part allocated in the central core of ENP. Although the BTB/POZ domain provides significant support (see ENP-∆Nterm), ENPs polarity is still realizable without this domain whereas the core is not dispensable for this (see ENP-∆NPH3_3). The C-terminal part is largely unnecessary for polarity but supports lateral accumulation of polar ENP. Its interaction with PINs, such as PIN2, might likely contribute to this accumulation and corroborates the view of a mutual support of ENP, MELs and PINs in polarity [25]. However, PINs alone might not represent the complete machinery for ENPs polarity because ENP-∆Cterm perfectly polarizes in PIN1/2 wild-type background although the strongest interaction with PIN2 occurs with its C-terminus. Together, ENP and MEL4 (representing MELs) carry the information for polarity mainly in their central core. The read out of this information depends on the tissue where they are expressed and is valid for either apical or basal localization.

### ENPs N-terminus, linker and central core participate in ENPs PIN supporting function

Partial deletions of ENP enabled us to identify the main region responsible for polarity. These deletions also lost functionality, indicating polar localization being a precondition for functionality. Considering the support of PIN activity, this was expected. Interestingly, point mutations of conserved residues showed, that the tolerance for alterations within the N-terminus, the linker and the central core seems to be high with respect to polarity, because the point mutations (except Y409E) displayed significant if not perfect polarity. We are aware that the latter is not an adequate phosphomimic since it is well known that glutamate (and even less aspartate) is unable to mimic either charge or the volume of pTyrosine [36]. The disturbed cellular distribution and degradation of ENP-Y409E might be caused by a severe structural impact in the helix (406D to 418E). Consequently, the “mild” exchange Y409A retains polarity and supports the view of the mentioned tolerance. Considering function, the significance of this region has to be refined as validated by incomplete restoration of ENP function in the *enp pid* double mutant. All mentioned point mutations significantly loose functional capability. Similarly, the *in frame* deletion of G407, L408 and Y409 lead to a complete amorphic loss-of-function allele [24]. The retention of polarity in these point mutations suggests that ENPs polarity is supported by more than one (or few) highly conserved residue. This notion is corroborated by the detected (FRET) contacts with the N- and C-terminus, the lateral accumulation by “addition“ of ENPs C-terminus as compared to the ENP-∆Cterm construct and the distribution of potential contact sites (BH scan) found along the entire ENP protein. In this context, the complete failure of MEL4/NPY4 constructs to restore any ENP-like function is indicative. The demonstrated basal vs. apical polarity in inner tissues vs. the epidermis respectively, reflects the evolutionary progress. A reasonable interpretation is, that MEL4/NPY4 (and likely the other MELs/NPYs as well) have retained sufficient similarity to ENP in the N-terminus, the linker and the central core to enable the cellular machinery to realise these polarities. However, the functional support of PIN function is more sensitive and is prevented by numerous dissimilarities in these regions. Thus, although the C-terminus possesses the major control on functionality, a structural integrity of the whole protein is required pointing to a functional role of the protein as a whole.

### ENPs C-terminus interacts with PINs and is an IDR whose function is critically affected by the modification of Ser^514^ and Ser^553^

Low pLDDT scores, as those given in ENPs aa471 to aa571, likely describe intrinsically disordered regions as opposed to well-defined autonomously foldable three-dimensional structures [30,58]. For instance, a very high number of regions with low pLDDT scores of the human proteome overlaps with regions of intrinsic disorder [30,59]. Furthermore, IDRs are not unstructured, they rather undergo disorder-to-order transitions (and vice versa) depending on special environmental and physiological conditions and take over important biological functions [35,60,61].

ENPs C-terminal sequence displays an additional feature found in some IDRs. Besides numerous serines and arginines, the C-terminus buries a repeating peptide motif SSSSSSRRRR (aa558-aa567). Such low-complexity regions are known in IDRs to form “collapsed globule“-assemblies as opposed to “extended coils“with alternating aa sequences [62,63].

Finally, IDRs are prominent for harbouring, in particular serine and threonine, phosphorylation sites [36,38], which can be linked to folding and regulatory switches [e.g., 64]. Such functional phosphorylation sites appear to be present in the C-terminus of ENP. Alterations of both tested phosphorylation sites (S514 and S553) likely impact on ENPs function in terms of restoration of PIN1 supporting ENP function in *enp pid*.

**Fig 9:**
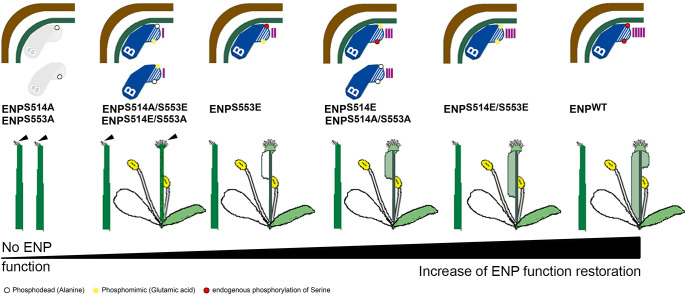
ENP C-term phosphomimic variants and *enp* rescue. Indicated is the flower development in *enp pid* plants by ENP constructs with modified C-termini. To the left are construct variants with no or inferior restoration of ENP function in terms of flower organ development and seed production. Note, the double phospho-dead construct is in the middle of this series. The grade of rescue is symbolized by flower-less, blind stems and irregular flowers without gynoecia (no carpels), gynoecia with empty carpels (white, no seed development) and gynoecia with increasing carpel tissue and increasing seed set (Fig 4A). All classes developed blind stems though with decreasing frequency towards the right (non-altered full length ENP^WT^). The better and more frequent flower development was paralleled by an increasing number of stems carrying bracts/cauline leaves (Fig 4A). Note that stigmatic papillae could develop even on blind stems and tissue replacing gynoecia (arrowheads).

Considering the data, the effectivity of ENPs functional restoration varies between no and full rescue depending on the status of S514 and S553 (Fig 9). The spectrum begins (no functional restoration) with one free site for phosphorylation combined with one fixed alanine (no charge), then to one glutamic acid (charge) and one serine (free for phosphorylation), matched by two fixed alanines (equal charge) or followed by two glutamic acids (equal charge) and reaches full functional restoration in the full-length constructs (Figs 9 and 4A). The non-modified full length constructs possess “free“ (S514/S553) sites, which are eventually phosphorylated by endogenous kinases. However, even full-length constructs do not reach the seed set of the *pid* single mutant with a natural ENP wild-type copy (Fig 4A). The fused GFP proteins might cause slight differences to the *pid* single mutant with respect to (residual) flower formation and fertility. Considering the shown interaction of the C-terminus with PIN2 it is likely, that biased and non-biased modifications respectively might be structurally detrimental or sub-optimal for this interaction as compared to full wild-type phosphorylation. Our observations provide starting points for further in-depth structural analyses on modification of ENPs C-terminal IDR. An interesting idea is, whether the instability of the single phospho-dead constructs could indicate a side effect. The detrimental effects of incompletely phosphorylated ENP might guarantee kinase activity until phosphorylation is completed, like a safeguard or counting mechanism. Taking together, the C-terminus of ENP likely represents an intrinsic disordered region, which is essential for ENPs activity and can be modulated by modification of selected target sites. This is at least partly attributed to a PIN-interaction, whose strength depends on its phosphorylation status.

**Fig 10:**
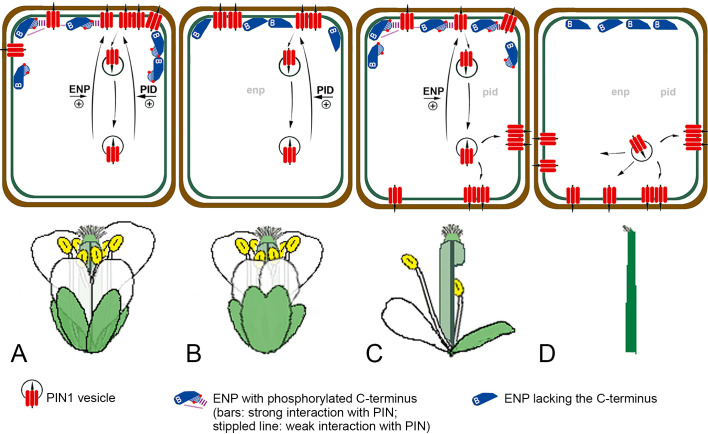
The independent impact of ENP on support of PIN1 function. The scheme models the contribution of ENP and PID activity on PIN1 function and development of flower structures (other factors excluded). Our model implies a support at least by partial support of PIN1 apical polarity based on the finding that PIN1 changes apical to lateral and basal polarity in the original *enp-1* mutant allele [16]. It also takes into account (residual) apical PIN1 polarity in *pid* single mutant embryos [16] and considers the impact of *pid* mutations in adult plants [13]. A) In wild-type both activities ensure optimal polarity of the PIN1 protein population. PIN1 vesicles are efficiently recruited to the apical end of epidermal cells. The model assumes that the interaction of ENPs C-terminus with PINs contributes to this polarity. B) This contribution is lost in the truncated *enp-1* single mutant although the truncated protein is still apically localized because PID activity still sustains apical polarization [13,16] of sufficient but less PIN1 molecules, as shown in [22], for the development of most organs except sepals, which are often fused. This also allows significant seed set. C) In *pid* single mutants the major impact on PIN1 polarity is absent, but ENPs contribution sustains residual flower development including seed set. The correct phosphorylation of ENP (C-terminus) ensures optimal activity. PIN1 is increasingly localized to basal and lateral plasma membrane regions. D) In *enp pid* double mutants both activities are absent and PIN1 is only laterally and basally localized (default basal GNOM transport). Leaf and flower organs on stems (as well as cotyledons) are completely absent in these plants.

The accumulated data of this and previous studies delineate a model of how ENP independently impacts on organogenesis by supporting PIN function, which is substantiated by several observations (Fig 10). PID activity has a major impact on PIN1 polarity and activity [13] even in an *enp* mutant background [16] (Fig 10A and 10B). ENPs contribution is then visible by two effects. On the molecular level, the abundance of PIN1 carriers is reduced at the PM [22]. This has mild but detectable consequences on the developmental level as seen by fused sepal organs [16]. In the *pid*-background (Fig 10C), residual (polar) PIN1 maintains low auxin flux [16]. This is likely enabled by the interaction of PINs and ENPs (phosphorylated) C-terminus and results in plants with (partly) *pin*-formed stems and stems with abnormal, fertile flowers (this study). In *pid heart stage* embryos the PIN1 population is distributed on apical, lateral as well as basal regions of the PM [16]. In *enp pid* double mutants (Fig 10D), the cellular PIN1 population has completely shifted to lateral and basal PM regions and all plants only develop blind stems [16]. In this study, the ENP-ΔCterm construct mimics the original *enp* allele and suggests that the lateral/basal shift of PIN1 is due to the absence of ENPs C-terminus while ENP remains apically localized. This model implies that *pin pid* [17] resemble *enp pid* double mutants. Some of the aforementioned factors, whose mutants result in cotyledon- and flower-less phenotypes in the *pid* background, might also contribute to the PID-independent input.

## Materials and methods

### Plant material, growth conditions and seedling culture

*Arabidopsis thaliana* (ecotype L*er*-0), EMS-induced single/double mutants and transgenic construct lines were grown according to conventional procedures under continuous light or 12 hrs light/12 hrs dark cycles (details in S1 Text, p1).

### Cloning and site directed mutagenesis, deletion and domain swap constructs

Briefly, *ENP* and *MEL4* wild-type full-length cDNA clones (pda08292 and pda10515, Riken Bio Resource Center, Japan) were used as starting material for further cloning by conventional restriction-ligation or Gateway technology (Thermo Fisher Sc.). For deletion, domain swap and site-directed mutagenesis constructs, appropriate primers extended with restriction sites recombination sites were used (see S1 Text). For site-directed mutagenesis the Quick Change II (Agilent) or the Q5 Site-Directed mutagenesis Kit (NEB) according to the supplier’s instructions were used (details in S1 Text, p2-7).

### Sequencing

We assessed critical regions on all levels of cloning and (after) transformation in *E. coli*, *A. tumefaciens* and *A. thaliana* with appropriate primers by sequencing (EUROFINS sequencing services; details in S1 Text, p8-9).

### Plant transformation

Plants were transformed according to conventional methods using *Agrobacterium tumefaciens* strain GV3101 (details in S1 Text, p9).

### Chemicals and pharmacological studies

Seedlings were treated with 10 mM PBA (MERK) as described [33] and with FM4–64 (1,7 µM – 2 µM; ThermoFisher Sc.) for 2–5 min, washed in water and processed for Imaging and/or FLIM-FRET analysis (details in S1 Text, p9).

### Immunocytochemistry

PIN1 localization in embryos transgenic for 35Sp:EGFP-ENP used PIN1 primary rabbit antibody incubation (1:1000; 4h, 37°C) and secondary rabbit Cy3-Antibodies (BSA/PBS for 3.5h at 37°C; Jackson ImmunoResearch/USA supplied by Dianova/Hamburg). After repeated washes with PBS and H_2_0 the embryos were embedded in Citifluor antifadent mounting medium and covered with a coverslip, stored at 4°C or −20°C or immediately processed for imaging (details in S1 Text, p10).

### In situ hybridization

Verification of mRNA patterns in embryos was done as previously described [4,16]. Details are given in S1 Text, p11-14).

### Confocal laser scanning microscopy (CLSM) and FRAP analysis

Imaging was done with Olympus FV1000 or FV3000 and 20X/0.75 NA air Plan-Apochromat or 63X/1.2 NA Plan-Apochromat water objective and TCS SP8 Leica equipped with a 63XW/NA 1.2 Plan-Apochromat water objective using the corresponding company software. Imaging used excitation laser lines 488 nm Argon, 488 nm diode, 515 nm diode or 561 nm diode lasers and appropriate detection (windows) Olympus PMT or GAsP detectors or Leica TCS SP8 HyD or PMT detectors. While HighVoltage detector setting was adjusted according to signal strength, non-linear signal amplification was not performed. Also, other than zero threshold setting (“Offset“) was regularly avoided.

FRAPs were performed with the TCS SP8 CLSM with 20 µm (40 µm and 60 µM) bleach spot diameters. After bleaching at high intensity with the 488 nm Argon laser, fluorescence intensities of the same and unbleached control regions (same spot size) were measured at different time intervals and normalized according to I_n_ = (I_t_-I_0_)/(I_I_ - I_0_), where I_t_ is the value of the recovered fluorescence intensity at any time t, I_0_ is the first post-bleach fluorescence intensity and I_I_ is the initial (pre-bleach) fluorescence intensity. Exact description of the procedures can be found in S1 Text, p14-15 and p17-19).

### Measurement of GFP-signals at the PM

The extension of GFP signal in transgenic plants carrying ENP-∆Cterm and full length ENP construct respectively, a special procedure was applied (“smile“analysis). Cells within the epidermal and cortex region were measured, if at least five were suitable for measurements. For each cell three different measurements were made. The first of the apical membrane, the second of the residual membrane and the third measurement was of the whole length of the GFP signal (S10 Fig). Then, GFP-signal lengths over apical membrane lengths, and GFP-signal length over total cell circumference (= apical + residual membrane length) were calculated and subjected to individual *t-*Tests.

A further series of measurements quantified and compared the signal intensity (as indirect measure of ENP-GFP fusion protein abundance) of various ENP constructs with full length, deletions and phosphomimic/phospho-dead mutations in ENPs C-terminus. Therefore, CLSM analyses were performed with appropriate standardized parameters. A Corrected Total Cell Fluorescence (CTCF) was determined based on the images obtained. The CTFC is an integrated signal density, i.e., the area of selected cell multiplied with the mean fluorescence of background readings. For details of these procedures see S1 Text, p15-17.

### Fluorescence lifetime imaging microscopy (FLIM) and förster resonance energy transfer (FRET) measurement

The lifetimes (τ) of donor Fluorophores (EGFP, GFP6) without and after non-radiative energy transfer to acceptor molecules (FM4–64, mCherry) were measured with the aid of a PicoQuant-Kit for Time Correlated Single Photon Counting (TCSPC) connected to a FV3000 Olympus CLSM following PicoQuant instructions. Energy transfer efficiency (E) was calculated according to: E = 1 − *τ*_DA_/*τ*_D_, where *τ*_**DA**_ is the fluorescence lifetime of the donor in presence of the acceptor and ***τ***_**D**_ is the fluorescence lifetime of the donor alone. For approximation of the distance (r) between (E)GFP and mCherry pairs we took R_0_ (the Förster distance at 50% energy transfer) from the “FPbase FRET Calculator (at https://www.fpbase.org/fret/) and used the equation for E expressed as E = R_0_^6^/(r^6^ + R_0_^6^) [56]. For details see S1 Text, p17-24.

## Supporting information

S1 Text Materials and methods. (DOCX)

S1 Table Quantification of organs in Ler_0 vs ENP constructs. (PDF)

S1 Fig ENP molecular structure. (PDF)

S2 Fig Clustal comparison of ENP & MELs. (PDF)

S3 Fig AIUPred analysis of ENP mutants. (PDF)

S4 Fig Pyrosequencing of ENP mutants. (PDF)

S5 Fig Onset of 35S-driven expression in embryos. (PDF)

S6 Fig ENP & MEL4 constructs. (PDF)

S7 Fig In situ hybridization with ENP. (PDF)

S8 Fig Cellular localization of MEL4 variants without and with PBA. (PDF)

S9 Fig Morphology of *laterne* & wild-type seeds. (PDF)

S10 Fig Analysis of lateral GFP signals of ENP-∆Cterm. (PDF)

S11 Fig FRAP analyses with 40 µM & 60 µM spots. (PDF)

S12 Fig ENP basic hydrophobic score plots. (PDF)

S1 Data Quantification of cotyledon and flower organs. (XLSX)

S2 Data Restoration of ENP function. (DOCX)

S3 Data Lateral extension of GFP signals. (XLSX)

S4 Data FRAPs 20 µM spot. (XLSX)

S5 Data FRAPs 40 µM spot. (XLSX)

S6 Data FRAPs 60 µM spot. (XLSX)

S7 Data PM Signal intensities. (XLSX)

S8 Data FRET ENP vs FM4–64. (XLSX)

S9 Data FRET ENP vsPIN2. (XLSX)
