# Supplementary material for: Distinct domains of ENHANCER OF PINOID hold information for its polarization required for auxin-mediated cotyledon and flower development in Arabidopsis
Source: PLoS Genet. 2025 Jun 23;21(6):e1011217. doi: 10.1371/journal.pgen.1011217 (PMC12201645; doi:10.1371/journal.pgen.1011217)
Supplement: S1 Table — (PDF) [file pgen.1011217.s002.pdf]

S1 Table: Quantification of cotyledon and flower organs in L*er*-0 and L*er*-0 transgenic with *35Sp:EGFP-ENP* and *35Sp:ENP-mGFP6.*

Construct	Plant no.	Cotyledons (mean value)	Flower no.	Sepals (mean value)	Petals (mean value)	Stamens (mean value)	Gynoecea (mean value)
L*er*-0	25	2	59	4	4	5,22	1
35Sp:EGFP-ENP	25	2	75	4	4	5,24	1
35Sp:ENP-mGFP6	25	2	75	4	4	5,38	1

See also S1 Data and S1 Text: Materials and Methods.
