# Supplementary material for: Distinct domains of ENHANCER OF PINOID hold information for its polarization required for auxin-mediated cotyledon and flower development in Arabidopsis
Source: PLoS Genet. 2025 Jun 23;21(6):e1011217. doi: 10.1371/journal.pgen.1011217 (PMC12201645; doi:10.1371/journal.pgen.1011217)
Supplement: S1 Fig — (PDF) [file pgen.1011217.s003.pdf]

Overview
ENP structures, domains, point mutations and deletions (comparison with MEL4/NPY4)

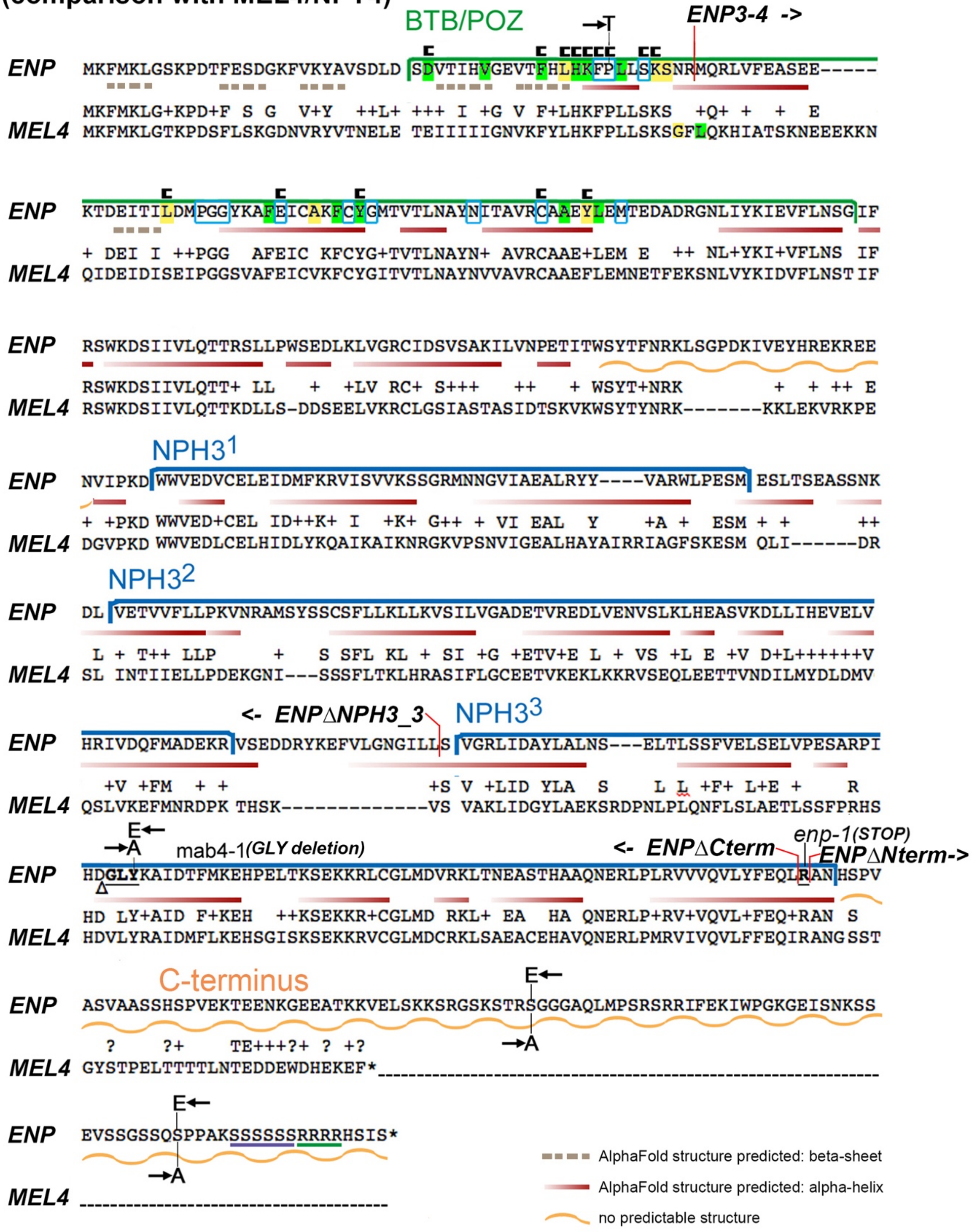

Length of ENP & MELs:

ENP	(At4g31820)	571 aas	position	R468 100% conserved in MELs
MEL4	(At2g23050)	481 aas	homologous	R450
MEL1	(At4g37590)	580 aas	-“-	R465
MEL2	(At5g67440)	579 aas	-“-	R476
MEL3	(At2g14820)	634 aas	-“-	R488

S1 Fig: Overview ENP protein structure and conservation

The Figure combines features described for the BTB/POZ domain (between aa1 and aa132) given in Stogios et al. [1] and the structure predictions by AlphaFold [2, 3].

The ENP sequence is compared with MEL4 (NPY4) indicating identical as well as similar ("+") aminoacids. The green bar above the aas indicates the core BTB/POZ domain [according to 1], which is then followed by sequences tentatively designated as "linker" region. The blue bars indicate the central NPH3-domain respectively, which is divided into three regions of higher similarity ($NPH3^1$ - $NPH3^3$) solely to point out the dissimilar regions in between where MEL4 is characterized by deletions.

The BTB/POZ domain includes the following information taken from Stogios et al. [1]. Yellow and green shaded aas have intermediate and high levels of conservation respectively. The latter also indicates positions that are similar in at least four of seven BTB/POZ families found in eukaryotes. Aas with blue frames indicate BTB/POZ-NPH3 specific signature sequences. The c-like symbols above the (highly) conserved residues indicate those, which in four non-plant families are contact sites for known protein-protein interactions.

The different lines below the sequence indicate information given by Alphafold [2, 3]. Stippled lines in light brown indicate beta-sheet structures whereas red lines give alpha-helical structures. Shorter red lines rather represent short helical turns. Wavy yellow lines indicate a shorter (aa181-aa205) and the long C-terminal tract (aa47-aa571) of intrinsic disorder. The majority of predicted beta-sheets and alpha-helices had very high per-residue confidence metrics (pLDDT > 90). The purple and green underlining at the very end of the protein points to a potential conformation resulting from the repeated S and R motif (for details see Text).

The figure also includes all deletions of ENP analyzed in this study (the -> arrows point to the sequences retained in the truncated protein). Also added are the point mutated

aas and the aas replacing them in this study. Note, that in S514 and S553 different combinations of double point mutations have been considered (see main text).

For comparison the length of ENP and all MELs is given at the bottom. The highly conserved aa R468 in the last AlphaFold predicted structure (alpha-helix) in ENP is given to indicate the length of the dissimilar C-termini of MELs beyond this site.

Literature

1. Stogios PJ, Downs GS, Jauhal JS, Nandra SK, Priv GG (2005). Sequence and structural analysis of BTB domain proteins. Genome Biology 2005, 6: R82.
2. Jumper J, Evans R, Pritzel A, Green T, Figurnov M, Ronneberger O et al. (2021) Highly accurate protein structure prediction with AlphaFold. Nature 596: 583-589.
3. Varadi M, Anyango S, Deshpande M, Nair S, Natassia C, Yordanova G et al. (2021) AlphaFold protein structure Database: massively expanding the structural coverage of protein-sequence space with high-accuracy models. Nucleic Acids Research 50: D439-D444.
