# Supplementary material for: Distinct domains of ENHANCER OF PINOID hold information for its polarization required for auxin-mediated cotyledon and flower development in Arabidopsis
Source: PLoS Genet. 2025 Jun 23;21(6):e1011217. doi: 10.1371/journal.pgen.1011217 (PMC12201645; doi:10.1371/journal.pgen.1011217)
Supplement: S2 Fig — (PDF) [file pgen.1011217.s004.pdf]

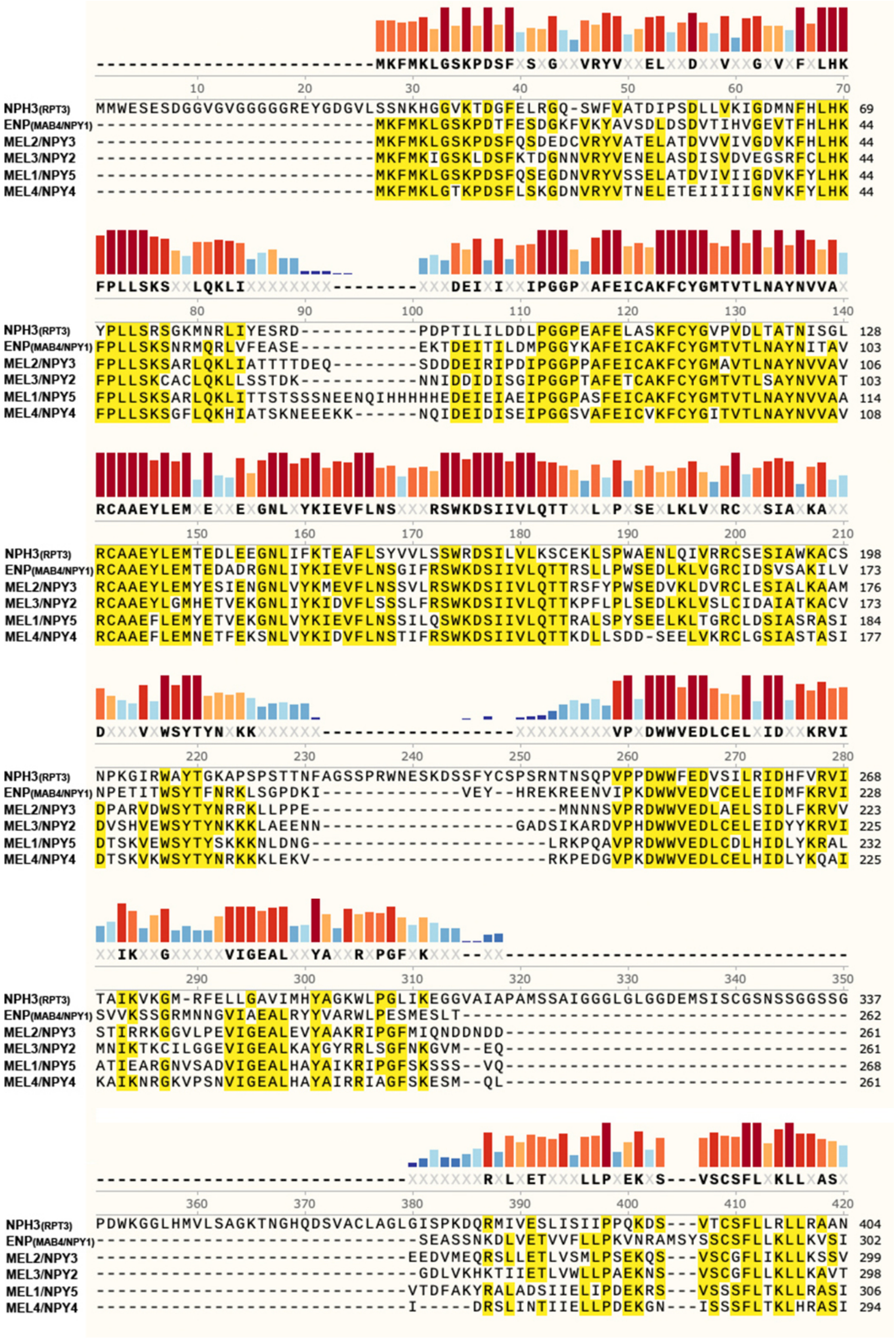

--------------------------MKFMKLGSKPDSFXSXGXXVRYVXXELXXDXXVXXGXVXFXLHK
10 20 30 40 50 60 70
NPH3(RPT3) MMWESESDGGVGVGGGGGREYGDGVLSSNKHGGVKTDGFELRGQ-SWFVATDIPSDLLVKIGDMNFHLHK 69
ENP(MAB4/NPY1) --------------------------MKFMKLGSKPDTFESDGKFVKYAVSDLDSDVTIHVGEVTFHLHK 44
MEL2/NPY3 --------------------------MKFMKLGSKPDSFQSDEDCVRYVATELATDVVVIVGDVKFHLHK 44
MEL3/NPY2 --------------------------MKFMKIGSKLDSFKTDGNNVRYVENELASDISVDVEGSRFCLHK 44
MEL1/NPY5 --------------------------MKFMKLGSKPDSFQSEGDNVRYVSSELATDVIVIIGDVKFYLHK 44
MEL4/NPY4 --------------------------MKFMKLGTKPDSFLSKGDNVRYVTNELETEIIIIIGNVKFYLHK 44
FPLLSKSXXLQKLIXXXXXXXX--------XXXDEIXIXXIPGGPXAFEICAKFCYGMTVTLNAYNVVAX
80 90 100 110 120 130 140
NPH3(RPT3) YPLLSRSGKMNRLIYESRD-----------PDPTILILDDLPGGPEAFELASKFCYGVPVDLTATNISGL 128
ENP(MAB4/NPY1) FPLLSKSNRMQRLVFEASE-----------EKTDEITILDMPGGYKAFEICAKFCYGMTVTLNAYNITAV 103
MEL2/NPY3 FPLLSKSARLQKLIATTTTDEQ--------SDDDEIRIPDIPGGPPAFEICAKFCYGMAVTLNAYNVVAV 106
MEL3/NPY2 FPLLSKCACLQKLLSSTDK-----------NNIDDIDISGIPGGPTAFETCAKFCYGMTVTLSAYNVVAT 103
MEL1/NPY5 FPLLSKSARLQKLITTSTSSSNEENQIHHHHHEDEIEIAEIPGGPASFEICAKFCYGMTVTLNAYNVVAA 114
MEL4/NPY4 FPLLSKSGFLQKHIATSKNEEEKK------NQIDEIDISEIPGGSVAFEICVKFCYGITVTLNAYNVVAV 108
RCAAEYLEMXEXXEXGNLXYKIEVFLNSXXXRSWKDSIIVLQTTXXLXPXSEXLKLVXRCXXSIAXKAXX
150 160 170 180 190 200 210
NPH3(RPT3) RCAAEYLEMTEDLEEGNLIFKTEAFLSYVVLSSWRDSILVLKSCEKLSPWAENLQIVRRCSESIAWKACS 198
ENP(MAB4/NPY1) RCAAEYLEMTEDADRGNLIYKIEVFLNSGIFRSWKDSIIVLQTTRSLLPWSEDLKLVGRCIDSVSAKILV 173
MEL2/NPY3 RCAAEYLEMYESIENGNLVYKMEVFLNSSVLRSWKDSIIVLQTTRSFYPWSEDVKLDVRCLESIALKAAM 176
MEL3/NPY2 RCAAEYLGMHETVEKGNLIYKIDVFLSSSLFRSWKDSIIVLQTTKPFLPLSEDLKLVSLCIDAIATKACV 173
MEL1/NPY5 RCAAEFLEMYETVEKGNLVYKIEVFLNSSILQSWKDSIIVLQTTRALSPYSEELKLTGRCLDSIASRASI 184
MEL4/NPY4 RCAAEFLEMNETFEKSNLVYKIDVFLNSTIFRSWKDSIIVLQTTKDLLSDD-SEELVKRCLGSIASTASI 177
DXXXVXWSYTYNXKKXXXXXX------------------XXXXXXXXXVPXDWWVEDLCELXIDXXKRVI
220 230 240 250 260 270 280
NPH3(RPT3) NPKGIRWAYTGKAPSPSTTNFAGSSPRWNESKDSSFYCSPSRNTNSQPVPPDWWFEDVSILRIDHFVRVI 268
ENP(MAB4/NPY1) NPETITWSYTFNRKLSGPDKI-------------VEY--HREKREENVIPKDWWVEDVCELEIDMFKRVI 228
MEL2/NPY3 DPARVDWSYTYNRRKLLPPE-----------------------MNNNSVPRDWWVEDLAELSIDLFKRVV 223
MEL3/NPY2 DVSHVEWSYTYNKKKLAEENN------------------GADSIKARDVPHDWWVEDLCELEIDYYKRVI 225
MEL1/NPY5 DTSKVEWSYTYSKKKNLDNG----------------------LRKPQAVPRDWWVEDLCDLHIDLYKRAL 232
MEL4/NPY4 DTSKVKWSYTYNRKKKLEKV----------------------RKPEDGVPKDWWVEDLCELHIDLYKQAI 225
XXIKXXGXXXXXVIGEALXXYAXXRXPGFXKXXX--XX--------------------------------
290 300 310 320 330 340 350
NPH3(RPT3) TAIKVKGM-RFELLGAVIMHYAGKWLPGLIKEGGVAIAPAMSSAIGGGLGLGGDEMSISCGSNSSGGSSG 337
ENP(MAB4/NPY1) SVVKSSGRMNNGVIAEALRYYVARWLPESMESLT------------------------------------ 262
MEL2/NPY3 STIRRKGGVLPEVIGEALEVYAAKRIPGFMIQNDDNDD-------------------------------- 261
MEL3/NPY2 MNIKTKCILGGEVIGEALKAYGYRRLSGFNKGVM--EQ-------------------------------- 261
MEL1/NPY5 ATIEARGNVSADVIGEALHAYAIKRIPGFSKSSS--VQ-------------------------------- 268
MEL4/NPY4 KAIKNRGKVPSNVIGEALHAYAIRRIAGFSKESM--QL-------------------------------- 261
-----------------------------XXXXXXXRXLXETXXXLLPXEKXS---VSCSFLXKLLXASX
360 370 380 390 400 410 420
NPH3(RPT3) PDWKGGLHMVLSAGKTNGHQDSVACLAGLGISPKDQRMIVESLISIIPPQKDS---VTCSFLLRLLRAAN 404
ENP(MAB4/NPY1) ------------------------------SEASSNKDLVETVVFLLPKVNRAMSYSSCSFLLKLLKVSI 302
MEL2/NPY3 -----------------------------EEDVMEQRSLLETLVSMLPSEKQS---VSCGFLIKLLKSSV 299
MEL3/NPY2 ------------------------------GDLVKHKTIIETLVWLLPAEKNS---VSCGFLLKLLKAVT 298
MEL1/NPY5 -----------------------------VTDFAKYRALADSIIELIPDEKRS---VSSSFLTKLLRASI 306
MEL4/NPY4 -----------------------------I-----DRSLINTIIELLPDEKGN---ISSSFLTKLHRASI 294

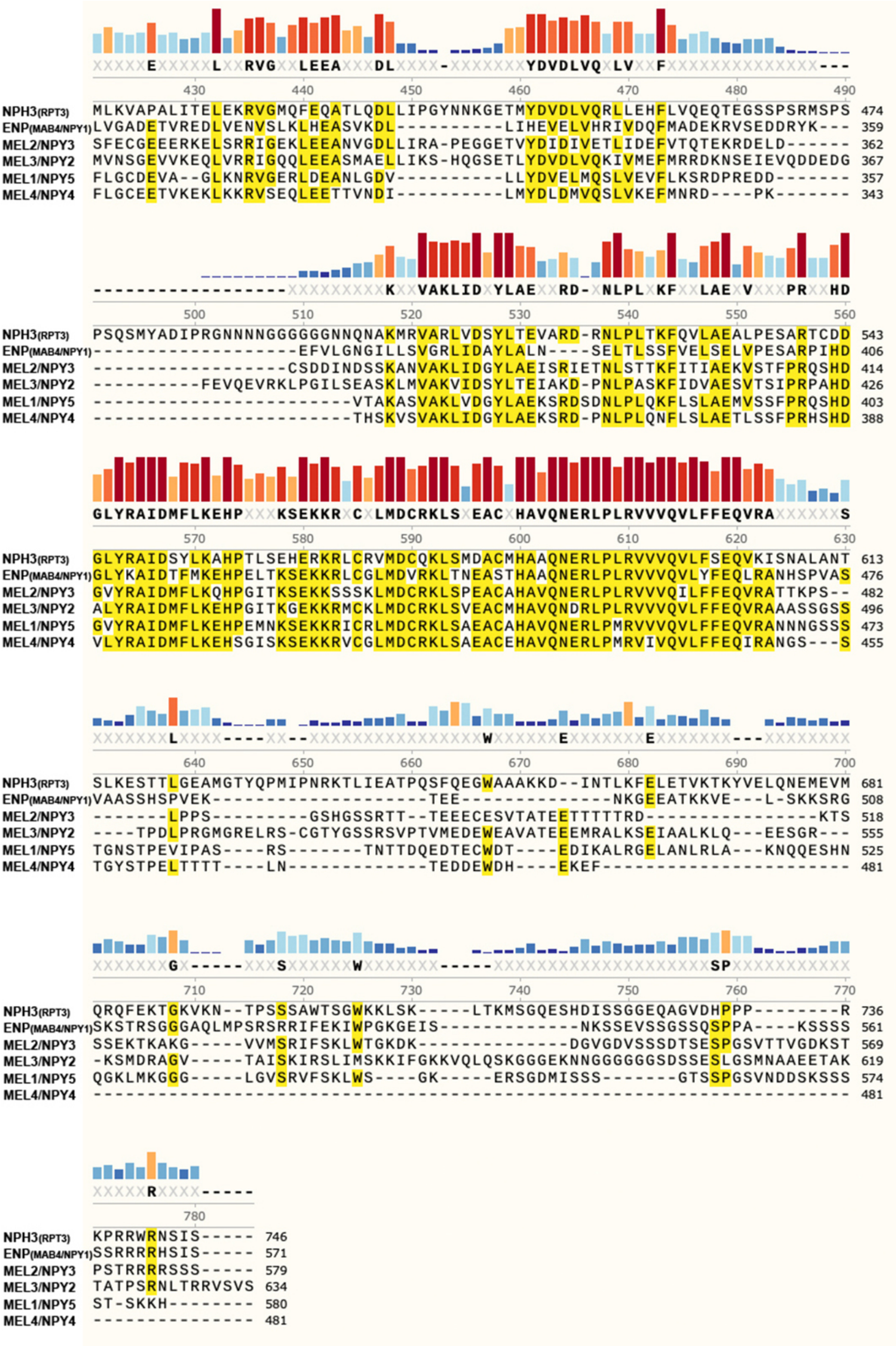

XXXXXEXXXXXLXXRVGXXLEEAXXXDLXXXX-XXXXXXXYDVDLVQXLVXXFXXXXXXXXXXXXXX---
430 440 450 460 470 480 490
NPH3(RPT3) MLKVAPALITELEKRVGMQFEQATLQDLLIPGYNNKGETMYDVDLVQRLLEHFLVQEQTEGSSPSRMSPS 474
ENP(MAB4/NPY1) LVGADETVREDLVENVSLKLHEASVKDL----------LIHEVELVHRIVDQFMADEKRVSEDDRYK--- 359
MEL2/NPY3 SFECGEEERKELSRRIGEKLEEANVGDLLIRA-PEGGETVYDIDIVETLIDEFVTQTEKRDELD------ 362
MEL3/NPY2 MVNSGEVVKEQLVRRIGQQLEEASMAELLIKS-HQGSETLYDVDLVQKIVMEFMRRDKNSEIEVQDDEDG 367
MEL1/NPY5 FLGCDEVA--GLKNRVGERLDEANLGDV----------LLYDVELMQSLVEVFLKSRDPREDD------- 357
MEL4/NPY4 FLGCEETVKEKLKKRVSEQLEETTVNDI----------LMYDLDMVQSLVKEFMNRD----PK------- 343
------------------XXXXXXXXXKXXVAKLIDXYLAEXXRD-XNLPLXKFXXLAEXVXXXPRXXHD
500 510 520 530 540 550 560
NPH3(RPT3) PSQSMYADIPRGNNNNGGGGGGNNQNAKMRVARLVDSYLTEVARD-RNLPLTKFQVLAEALPESARTCDD 543
ENP(MAB4/NPY1) -------------------EFVLGNGILLSVGRLIDAYLALN----SELTLSSFVELSELVPESARPIHD 406
MEL2/NPY3 ------------------CSDDINDSSKANVAKLIDGYLAEISRIETNLSTTKFITIAEKVSTFPRQSHD 414
MEL3/NPY2 ----------FEVQEVRKLPGILSEASKLMVAKVIDSYLTEIAKD-PNLPASKFIDVAESVTSIPRPAHD 426
MEL1/NPY5 ------------------------VTAKASVAKLVDGYLAEKSRDSDNLPLQKFLSLAEMVSSFPRQSHD 403
MEL4/NPY4 ------------------------THSKVSVAKLIDGYLAEKSRD-PNLPLQNFLSLAETLSSFPRHSHD 388
GLYRAIDMFLKEHPXXXKSEKKRXCXLMDCRKLSXEACXHAVQNERLPLRVVVQVLFFEQVRAXXXXXXS
570 580 590 600 610 620 630
NPH3(RPT3) GLYRAIDSYLKAHPTLSEHERKRLCRVMDCQKLSMDACMHAAQNERLPLRVVVQVLFSEQVKISNALANT 613
ENP(MAB4/NPY1) GLYKAIDTFMKEHPELTKSEKKRLCGLMDVRKLTNEASTHAAQNERLPLRVVVQVLYFEQLRANHSPVAS 476
MEL2/NPY3 GVYRAIDMFLKQHPGITKSEKKSSSKLMDCRKLSPEACAHAVQNERLPLRVVVQILFFEQVRATTKPS-- 482
MEL3/NPY2 ALYRAIDMFLKEHPGITKGEKKRMCKLMDCRKLSVEACMHAVQNDRLPLRVVVQVLFFEQVRAAASSGSS 496
MEL1/NPY5 GVYRAIDMFLKEHPEMNKSEKKRICRLMDCRKLSAEACAHAVQNERLPMRVVVQVLFFEQVRANNNGSSS 473
MEL4/NPY4 VLYRAIDMFLKEHSGISKSEKKRVCGLMDCRKLSAEACEHAVQNERLPMRVIVQVLFFEQIRANGS---S 455
XXXXXXXLXXXX----XX--XXXXXXXXXXXXXXXXWXXXXXXEXXXXXXXEXXXXXXX---XXXXXXXX
640 650 660 670 680 690 700
NPH3(RPT3) SLKESTTLGEAMGTYQPMIPNRKTLIEATPQSFQEGWAAAKKD--INTLKFELETVKTKYVELQNEMEVM 681
ENP(MAB4/NPY1) VAASSHSPVEK-------------------TEE---------------NKGEEATKKVE---L-SKKSRG 508
MEL2/NPY3 -------LPPS---------GSHGSSRTT--TEEECESVTATEETTTTTRD----------------KTS 518
MEL3/NPY2 ----TPDLPRGMGRELRS-CGTYGSSRSVPTVMEDEWEAVATEEEMRALKSEIAALKLQ---EESGR--- 555
MEL1/NPY5 TGNSTPEVIPAS----RS-------TNTTDQEDTECWDT----EDIKALRGELANLRLA---KNQQESHN 525
MEL4/NPY4 TGYSTPELTTTT----LN-------------TEDDEWDH----EKEF----------------------- 481
XXXXXXXGX-----XXXSXXXXXXWXXXXXXX-----XXXXXXXXXXXXXXXXXXXXSPXXXXXXXXXXX
710 720 730 740 750 760 770
NPH3(RPT3) QRQFEKTGKVKN--TPSSSAWTSGWKKLSK-----LTKMSGQESHDISSGGEQAGVDHPPP--------R 736
ENP(MAB4/NPY1) SKSTRSGGGAQLMPSRSRRIFEKIWPGKGEIS-------------NKSSEVSSGSSQSPPA----KSSSS 561
MEL2/NPY3 SSEKTKAKG-----VVMSRIFSKLWTGKDK--------------DGVGDVSSSDTSESPGSVTTVGDKST 569
MEL3/NPY2 -KSMDRAGV-----TAISKIRSLIMSKKIFGKKVQLQSKGGGEKNNGGGGGGSDSSESLGSMNAAEETAK 619
MEL1/NPY5 QGKLMKGGG-----LGVSRVFSKLWS----GK-----ERSGDMISSS-------GTSSPGSVNDDSKSSS 574
MEL4/NPY4 -- 481
XXXXXRXXXX-----
780
NPH3(RPT3) KPRRWRNSIS----- 746
ENP(MAB4/NPY1) SSRRRRHSIS----- 571
MEL2/NPY3 PSTRRRRSSS----- 579
MEL3/NPY2 TATPSRNLTRRVSVS 634
MEL1/NPY5 ST-SKKH-------- 580
MEL4/NPY4 --------------- 481

S2 Fig: Clustal Omega protein alignment of ENP and other NPH3 proteins.

Clustal Omega multiple protein sequence alignment of ENP (MAB4/NPY1), MEL2/NPY3, MEL3/NPY2, MEL1/NPY5, MEL4/NPY4 and NPH3 (RPT3) according to Madeira et al., 2024 [1]. The complete identity of all amino acids in a column is indicated by dark red and highest bar. From five to two identical amino acids the height of the bar decreases and changes color from red to dark/light orange and dark/light blue respectively. Intensity of color also depends on whether NPH3/RPT3 displays a residue identical to one of the other proteins. Height and (blue) color intensity of bars also considers amino acids in positions where one or more proteins lack a residue in the alignment.

Literature

1. Madeira F, Madhusoodanan N, Lee J, Eusebi A, Niewielska A, Tivey ARN, Lopez R, Butcher S (2024) The EMBL-EBI Job Dispatcher sequence analysis tools framework in 2024. Nucleic Acids Research 52: W521-W525.
