# Supplementary material for: Distinct domains of ENHANCER OF PINOID hold information for its polarization required for auxin-mediated cotyledon and flower development in Arabidopsis
Source: PLoS Genet. 2025 Jun 23;21(6):e1011217. doi: 10.1371/journal.pgen.1011217 (PMC12201645; doi:10.1371/journal.pgen.1011217)
Supplement: S3 Fig — (PDF) [file pgen.1011217.s005.pdf]

AIUPred Disorder Predictions for ENP and mutant variants

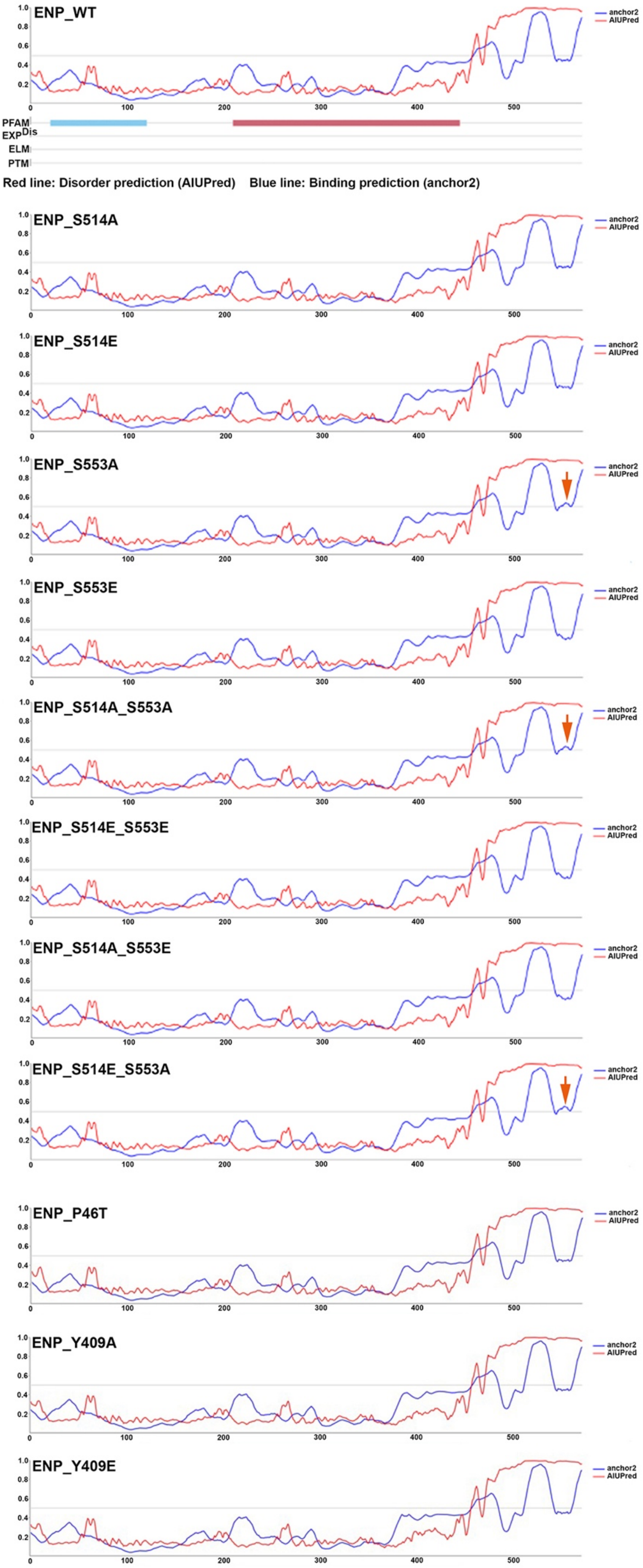

S3 Fig: AIUPred protein analysis of ENP variants

AIUPred is a novel version of IUPred, which incorporates deep learning techniques into the energy estimation framework allowing improved prediction of protein disorder [1], i. e. Intrinsic Disordered Regions (IDRs). The AIUPred-values range from 0 to 1 (1 means intrinsic disorder), which correspond for the probability of the given amino acid residue being part of a disordered region. PFAM indicates known protein domains as registered in the PFAM databank (http://pfam.xfam.org/). For the other characteristics there are no entries for ENP in this program. EXP^{Dis} stands for experimentally validated IDRs; PTM for known post-translational modifications and ELM for known motifs from the eukaryotic linear motif database (ELM). The ANCHOR program provides values for possible binding of particular residues/sequences. Note that this value is elevated whenever the residue S553 is replaced by 553A.

Literature

1. Erdös G, Doszranyi Z (2024) AIUPred: combining energy estimation with deep learning for the enhanced prediction of protein disorder. Nucleic Acids Research 52: W176-W181.
