# Supplementary material for: Distinct domains of ENHANCER OF PINOID hold information for its polarization required for auxin-mediated cotyledon and flower development in Arabidopsis
Source: PLoS Genet. 2025 Jun 23;21(6):e1011217. doi: 10.1371/journal.pgen.1011217 (PMC12201645; doi:10.1371/journal.pgen.1011217)
Supplement: S4 Fig — (PDF) [file pgen.1011217.s006.pdf]

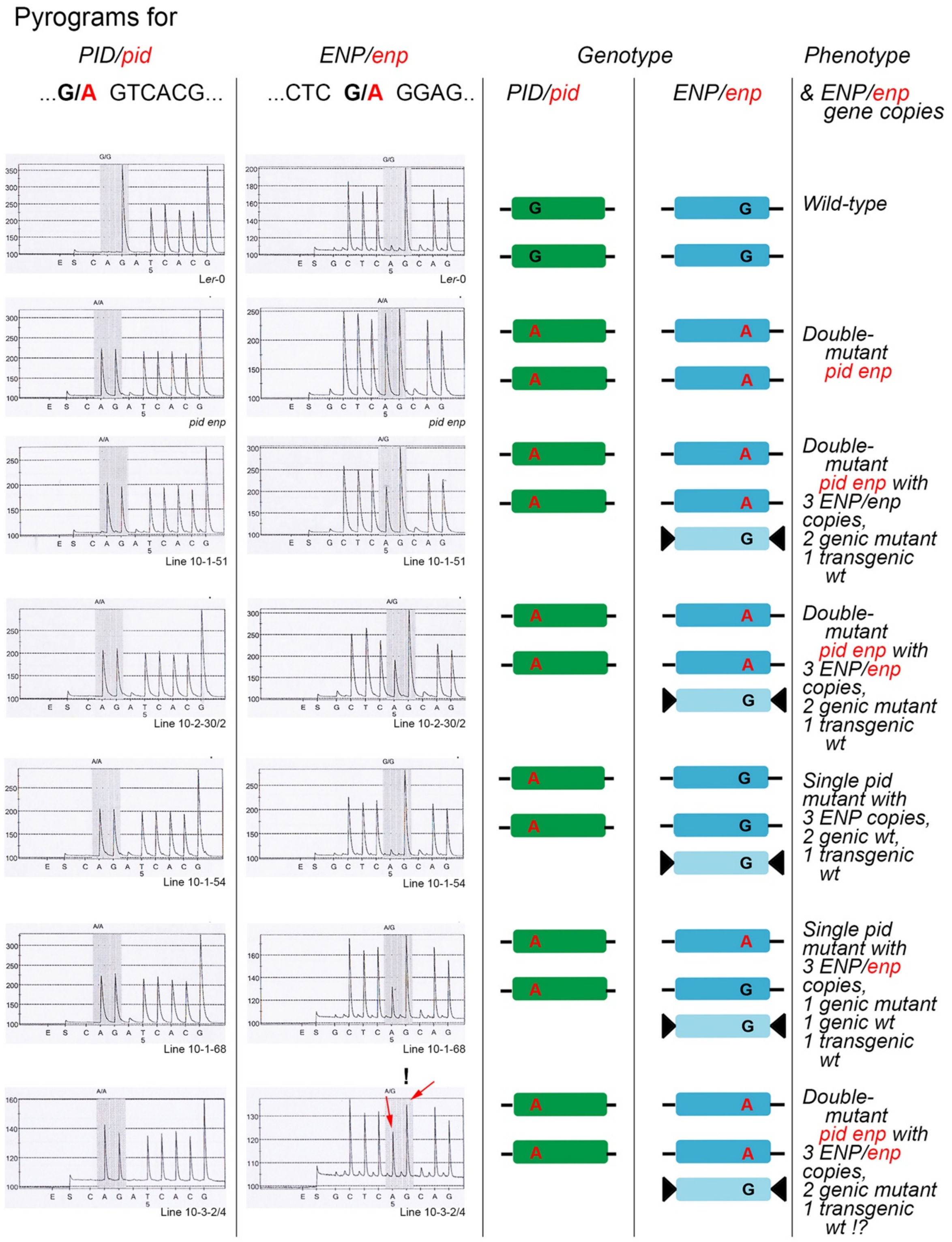

S4 Fig: Pyrosequencing of plants.

Critical sites, as indicated on top, of wild-type, *enp pid* double mutants and *pid* single mutants were sequenced by pyrosequencing as described [1]. Point mutations sequenced were those of the *pid-15* allele [1] and *enp-1* allele [1, 2]. Except the two

top-most, all plants carried the full length ENP transgene *35Sp:EGFP-ENP* (symbolized by inverted black arrowheads) and were resistant to BASTA/Phosphinotricin. The phenotypes of seedlings and adult plants were either wild-type (L*er*-0; normal developed plant), *pid* (three cotyledons and abnormal but fertile flowers) and *pid enp* (no cotyledons, and rescue of flower structures). Note the height of the peaks (quantitatively) in the detection window of the pyrogram for the *PID/pid* (left) and *ENP/enp* (right) wild-type/mutant base. The bottom-most individual is a case where the height and the proportion of the peaks was not satisfying. Genotypes and phenotypes indicated.

Literature

1. Treml BS, Winderl S, Radykewicz R, Herz M, Schweizer G, Hutzler P et al. (2005) The gene *ENHANCER OF PINOID* controls cotyledon development in the *Arabidopsis* embryo. Development 132: 4063-4074.
2. Furutani M, Kajiwara T, Kato T, Treml BS, Stockum C, Torres-Ruiz RA, Tasaka M (2007) The gene *MACCHI-BOU4/ENHANCER OF PINOID* encodes a NPH3-like protein and reveals similarities between organogenesis and phototropism on the molecular level. Development 134: 3849-3859.
