# Supplementary material for: Distinct domains of ENHANCER OF PINOID hold information for its polarization required for auxin-mediated cotyledon and flower development in Arabidopsis
Source: PLoS Genet. 2025 Jun 23;21(6):e1011217. doi: 10.1371/journal.pgen.1011217 (PMC12201645; doi:10.1371/journal.pgen.1011217)
Supplement: S5 Fig — (PDF) [file pgen.1011217.s007.pdf]

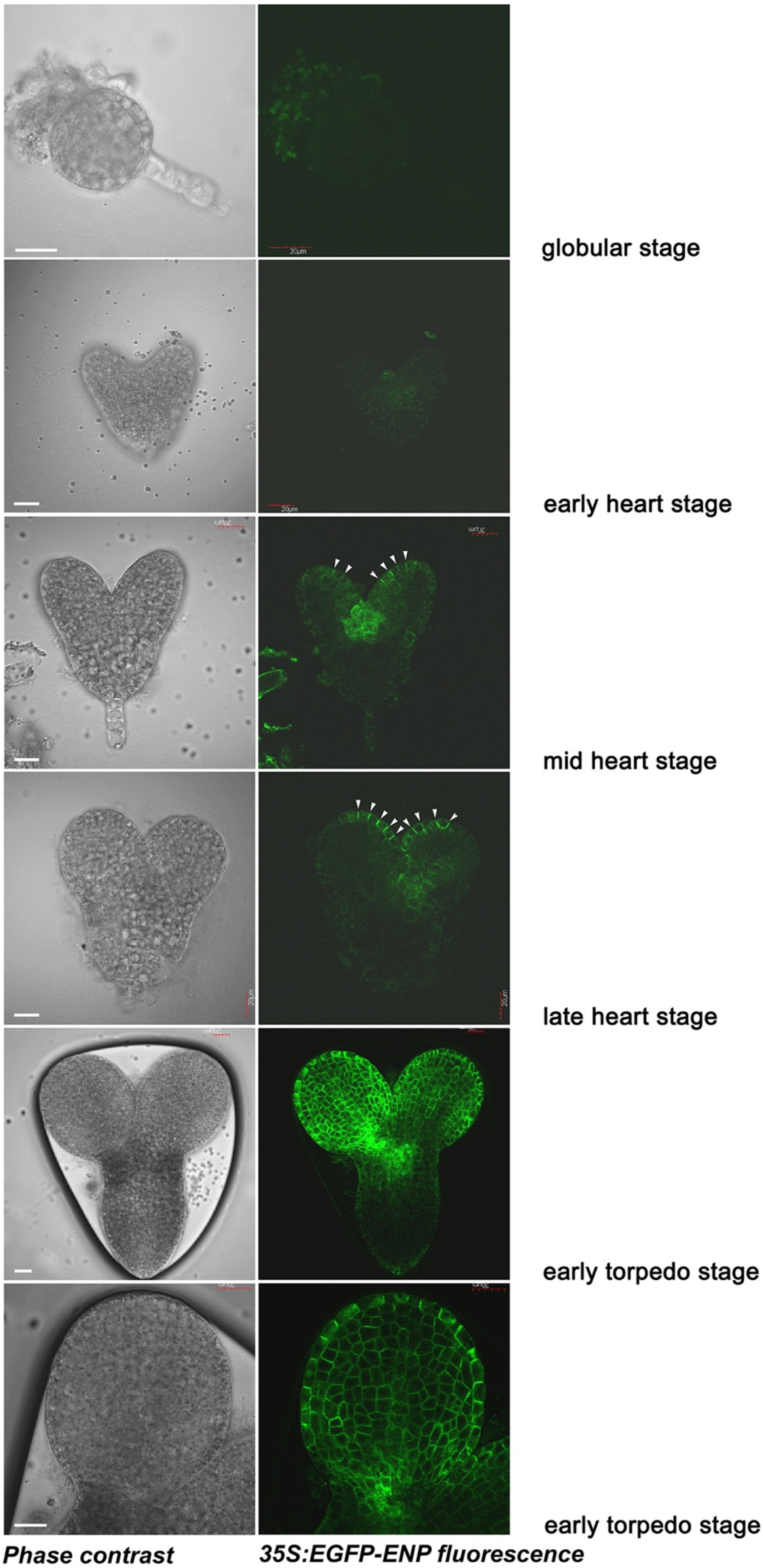

S5 Fig: Onset of ENP expression driven by the 35S promoter

Shown are from top to bottom as indicated: globular, early heart stage, mid heart stage, late heart stage, early torpedo stage (whole embryo and magnification of one cotyledon). A very weak signal is visible in the shoot apical meristem region in early heart stage. However as shown, cotyledon primordia do already form in this stage. In mid heart stage first polarized localization of EGFP-ENP is visible (white arrowheads). Scale bars: 20μM.
