# Supplementary material for: Distinct domains of ENHANCER OF PINOID hold information for its polarization required for auxin-mediated cotyledon and flower development in Arabidopsis
Source: PLoS Genet. 2025 Jun 23;21(6):e1011217. doi: 10.1371/journal.pgen.1011217 (PMC12201645; doi:10.1371/journal.pgen.1011217)
Supplement: S6 Fig — (PDF) [file pgen.1011217.s008.pdf]

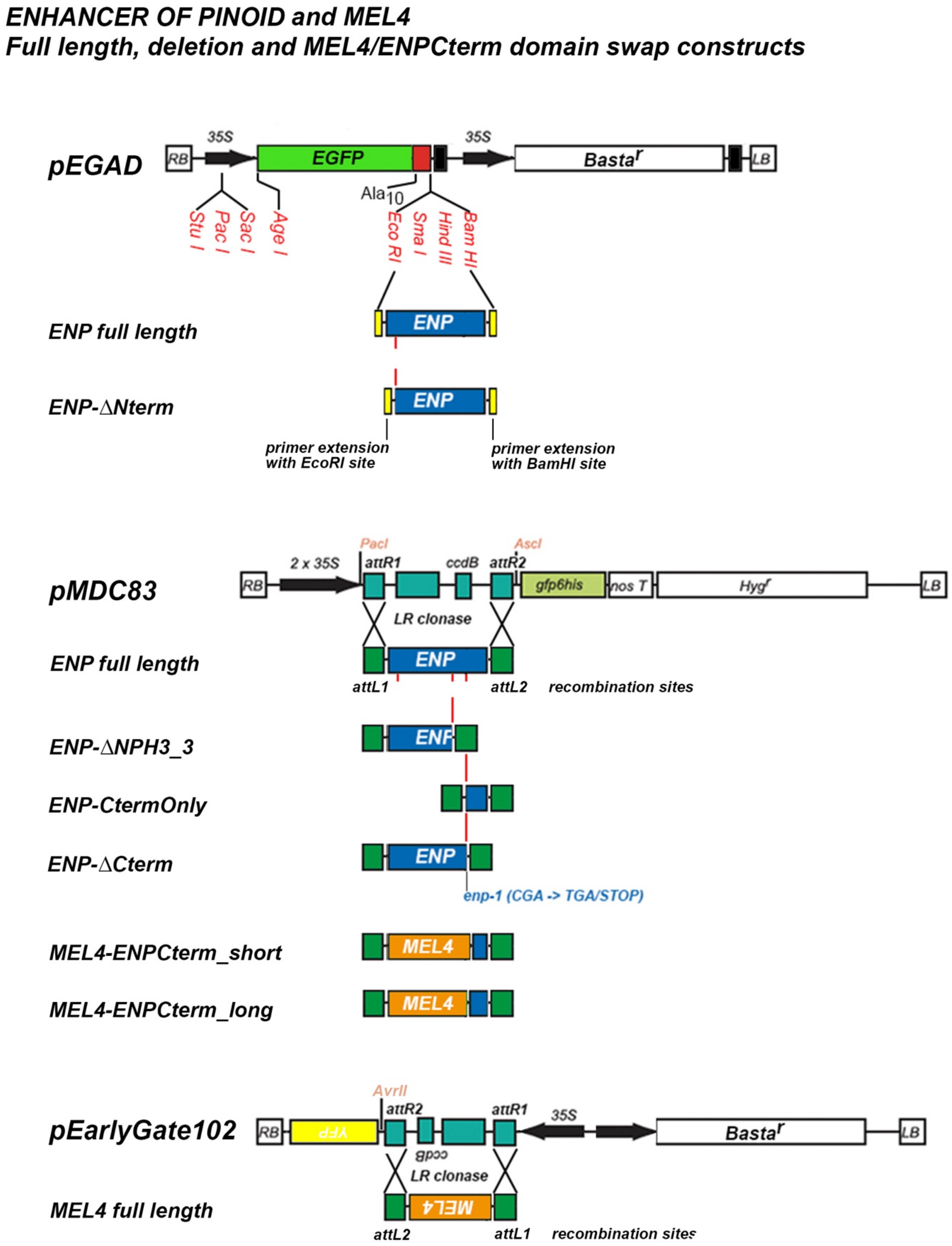

S6 Fig: Cloning of constructs

The figure gives a schematic (not to scale) overview of the full length and deletion constructs analyzed in this study. For N-terminal positioning via restriction-ligation of EGFP the vector pEGAD [1] was used. All other constructs were generated via

GATEWAY cloning. The ENP constructs with C-terminal GFP6 including those with point mutations were introduced into the pMDC83 vector [2]. The MEL4 full length construct was generated using the pEARLYGATE102 vector (for details see S1Text Materials and Methods).

Literature

1. Cutler SR, Ehrhardt DW, Griffitts JS, Somerville CR (2000) Random GFP::cDNA fusions enable visualization of subcellular structures in cells of *Arabidopsis* at a high frequency. Proc Nat Acad Sci USA 97 (7): 3718-3723 (doi.org/10.1073/pnas.97.7.3718).

2. Curtis MD, Grossniklaus U (2003) A Gateway Cloning Vector Set for High-Throughput Functional Analysis of Genes in Planta. Plant Physiol 133: 462-469 (doi: 10.1104/pp.103.027979).
