# Supplementary material for: Distinct domains of ENHANCER OF PINOID hold information for its polarization required for auxin-mediated cotyledon and flower development in Arabidopsis
Source: PLoS Genet. 2025 Jun 23;21(6):e1011217. doi: 10.1371/journal.pgen.1011217 (PMC12201645; doi:10.1371/journal.pgen.1011217)
Supplement: S7 Fig — (PDF) [file pgen.1011217.s009.pdf]

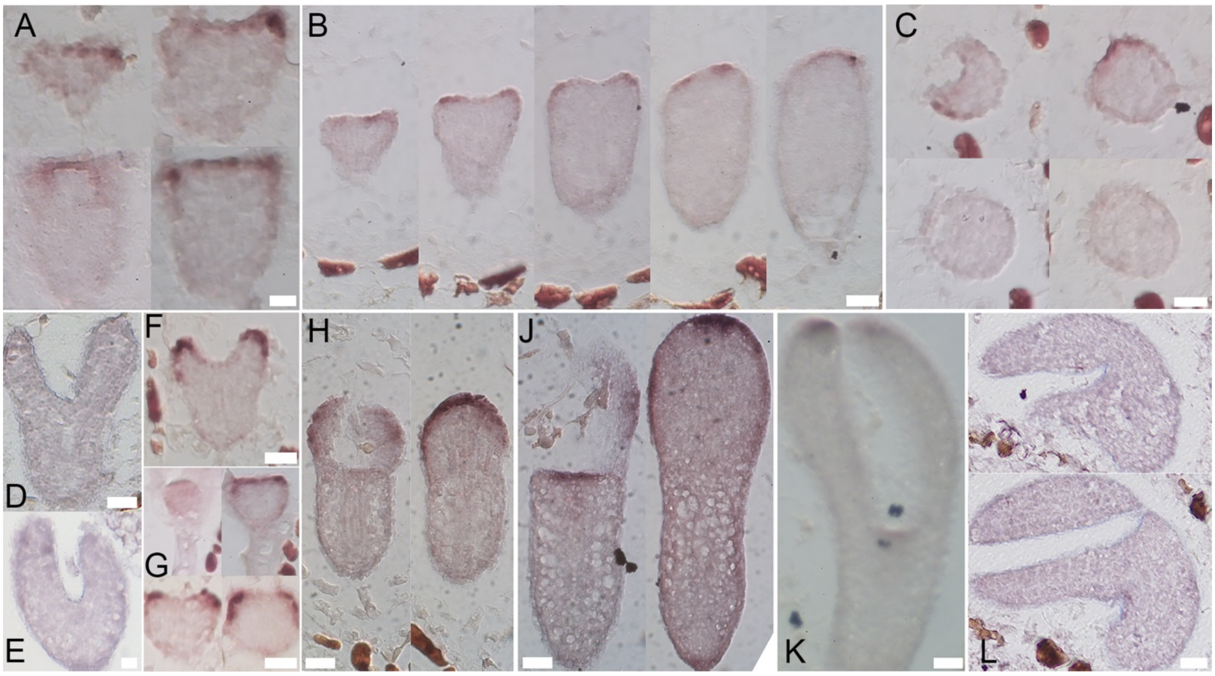

S7 Fig: ENP expression in embryos

In situ hybridizations with ENP antisense probe (A-C and F-K) and ENP sense probe as control (D, E and L). For method details see (S1 Text). A-C) Homozygous *enp pid* embryos lacking cotyledon primordia. A) Front view of mid heart stage embryo (series to be followed clockwise). B) Front view of mid-torpedo stage embryo (series from left to right). C) Top view on an *enp pid* embryo with the characteristic apical cavity, which can be seen in first three slices in B (series to be followed clockwise). Note the specific epidermal *ENP* expression signal in the apical region of the embryos. F-K) Wild-type embryos showing epidermal ENP expression in cotyledon primordia. F-J) Front view of mid heart stage (F),early globular, transition and two early heart stages (G, series to be followed clockwise), late heart/early torpedo stage (H) and mid torpedo stage (J). Side view of mid torpedo stage (K). Note the weak to strong signals in the epidermis of the shoot apical meristems and weak signals in the root tip outer layer. D, E and L) Control hybridizations with comparable embryo stages, i. e. late heart (D) and (mid) torpedo stages (E, L). Scale bars A: 10µM, B-L: 20µM.
