# Supplementary material for: Distinct domains of ENHANCER OF PINOID hold information for its polarization required for auxin-mediated cotyledon and flower development in Arabidopsis
Source: PLoS Genet. 2025 Jun 23;21(6):e1011217. doi: 10.1371/journal.pgen.1011217 (PMC12201645; doi:10.1371/journal.pgen.1011217)
Supplement: S8 Fig — (PDF) [file pgen.1011217.s010.pdf]

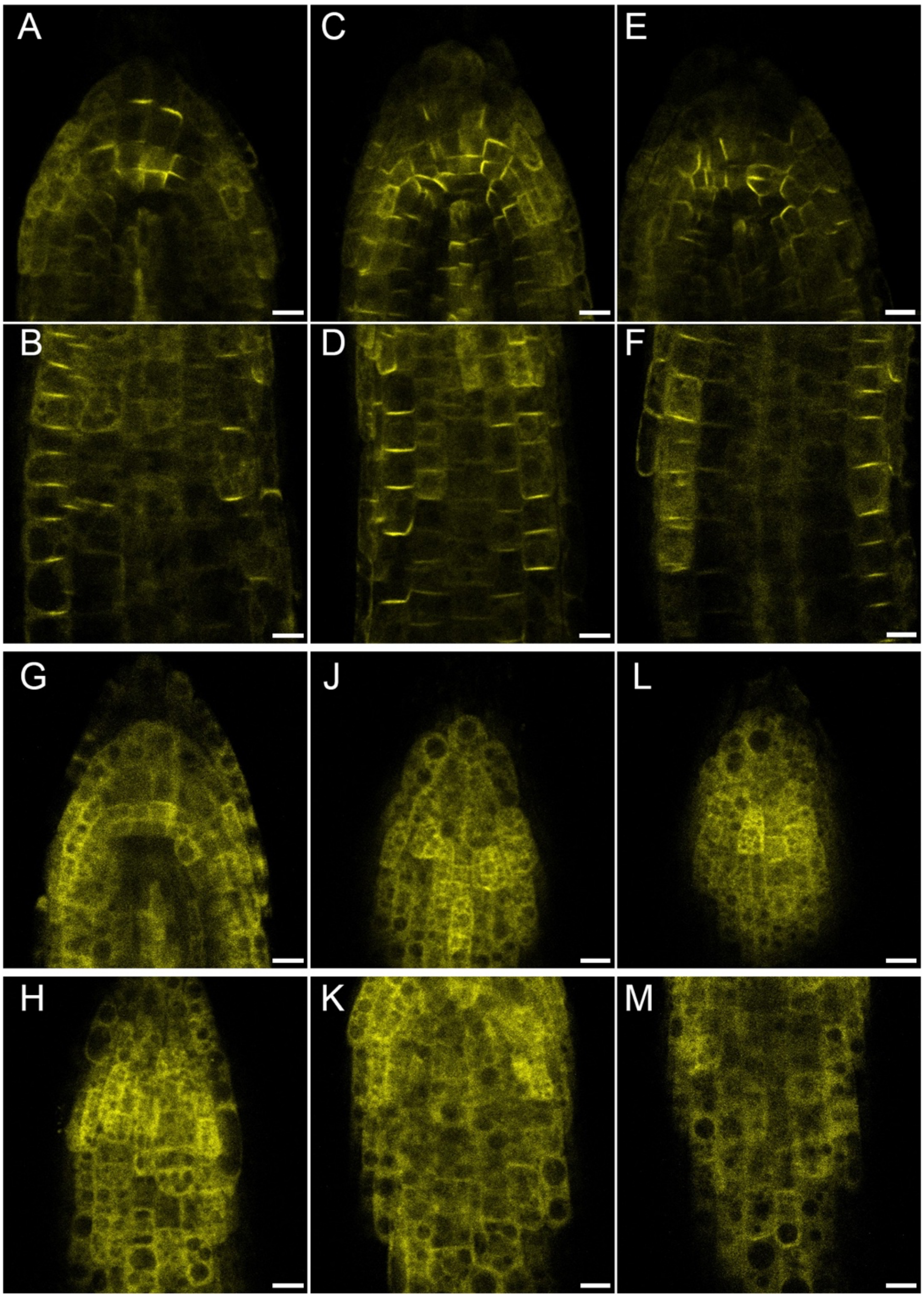

S8 Fig: MEL4 internalization upon PBA treatment

MEL4-EYFP shows the same response to phenylboronic acid (PBA, 10mM) as previously reported for ENP [1, 2].

A-F) Untreated control plants: root tip (A, C, E) and the same further up (B, D, F; meristematic region, focus on epidermis). G-M) Three representative plants treated with 10mM PBA. G, J, L: root tip; H, K, M: further up. Scale bars: 10µM.

Literature

1. Matthes M, Torres-Ruiz RA (2016) Boronic acid treatment phenocopies *monopteros* by affecting PIN1 membrane stability and polar auxin transport in *Arabidopsis thaliana* embryos. Development 143: 4053-4062.
2. Matthes M, Torres-Ruiz RA (2017) Boronic acids as tools to study (plant) developmental processes? Plant Signaling & Behavior 12:5, e1321190 (doi: 10.1080/15592324.2017.1321190).
