# Supplementary material for: Distinct domains of ENHANCER OF PINOID hold information for its polarization required for auxin-mediated cotyledon and flower development in Arabidopsis
Source: PLoS Genet. 2025 Jun 23;21(6):e1011217. doi: 10.1371/journal.pgen.1011217 (PMC12201645; doi:10.1371/journal.pgen.1011217)
Supplement: S9 Fig — (PDF) [file pgen.1011217.s011.pdf]

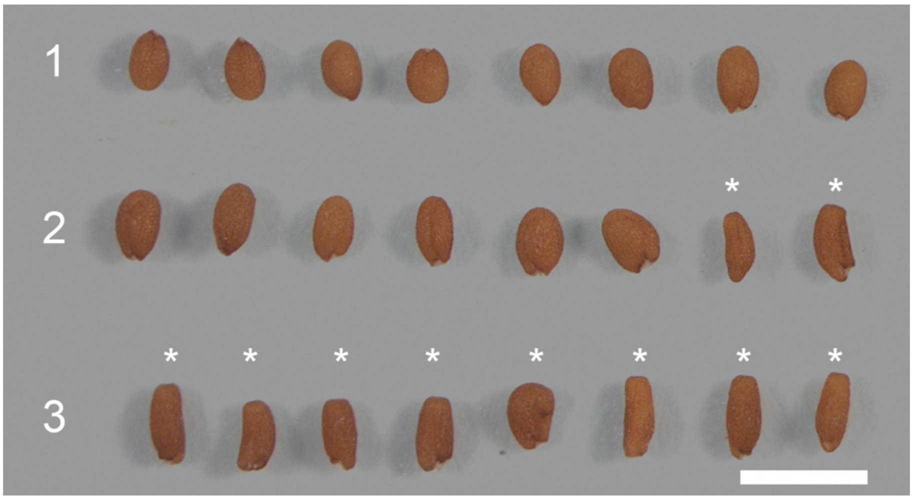

S9 Fig: Morphology of wild-type and homozygous mutant *enp pid* (*laterne*) seeds

Shown are seeds from three different plants. 1: Seeds from a wild-type *A. thaliana* (Ler-0) plant. The overall shape of the seed shell indicates the presence of cotyledons and the hypocotyl/root part. 2: Seeds of a plant with the genotype *enp pid/enp.* +. The two right-most seeds display a comma-like shape due to the missing cotyledons because the seed shell wraps closely around the remaining cotyledon-less embryo. 3: Seeds of a homozygous *enp pid/enp pid* plant which carried a *35Sp:ENP-GFP6* construct. The complete pedigree of this plant displays the *laterne*-like seed shape. Stars above seeds indicate the *enp pid/enp pid* (*laterne*) seed shape morphology. Scale bar: 1mm.
