# Supplementary material for: Distinct domains of ENHANCER OF PINOID hold information for its polarization required for auxin-mediated cotyledon and flower development in Arabidopsis
Source: PLoS Genet. 2025 Jun 23;21(6):e1011217. doi: 10.1371/journal.pgen.1011217 (PMC12201645; doi:10.1371/journal.pgen.1011217)
Supplement: S10 Fig — (PDF) [file pgen.1011217.s012.pdf]

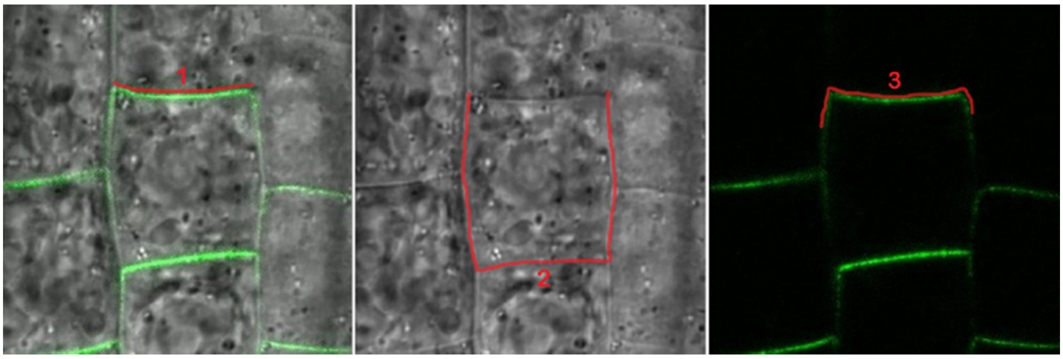

S10 Fig: Analysis of distribution of polar ENP

For distribution of ENP-localization within *Arabidopsis thaliana* cells, different seedlings were measured for ENP-GFP6 and ENP∆Cterm-GFP6. Only cells within the epithelial and cortex region were measured. For each cell three different measurements were made. The first for the apical membrane length (left: red line 1), the second of the residual membrane (middle: red line 2) and the third measurement for the whole length of the GFP signal (right: red line 3). The significance of differences with t-Test.
