# Supplementary material for: Distinct domains of ENHANCER OF PINOID hold information for its polarization required for auxin-mediated cotyledon and flower development in Arabidopsis
Source: PLoS Genet. 2025 Jun 23;21(6):e1011217. doi: 10.1371/journal.pgen.1011217 (PMC12201645; doi:10.1371/journal.pgen.1011217)
Supplement: S11 Fig — (PDF) [file pgen.1011217.s013.pdf]

A

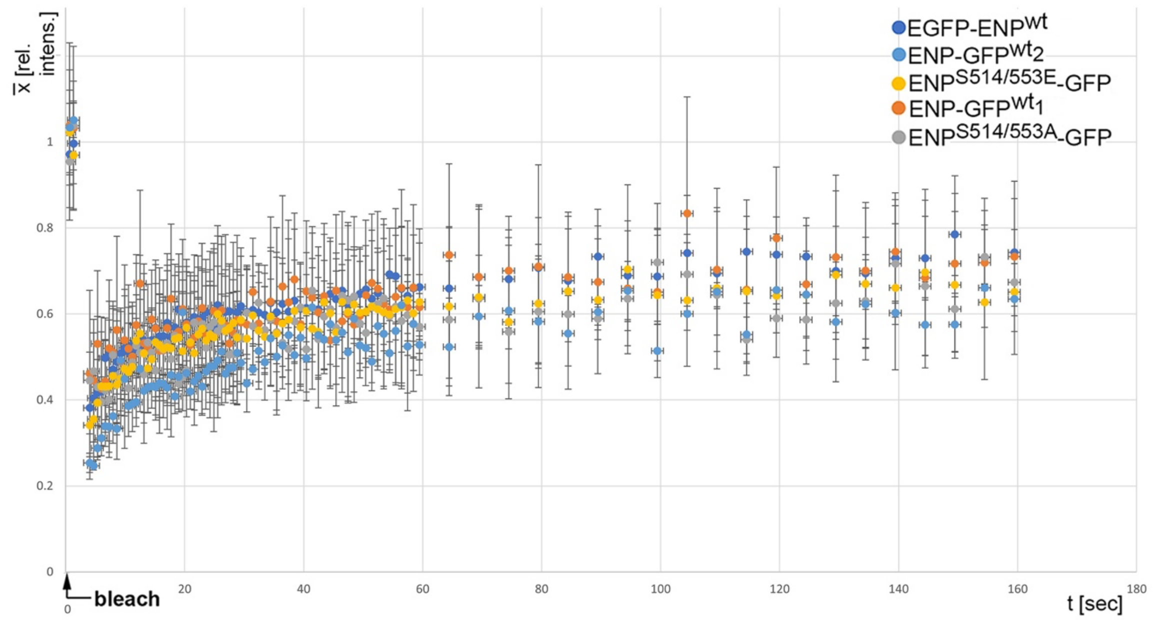

B

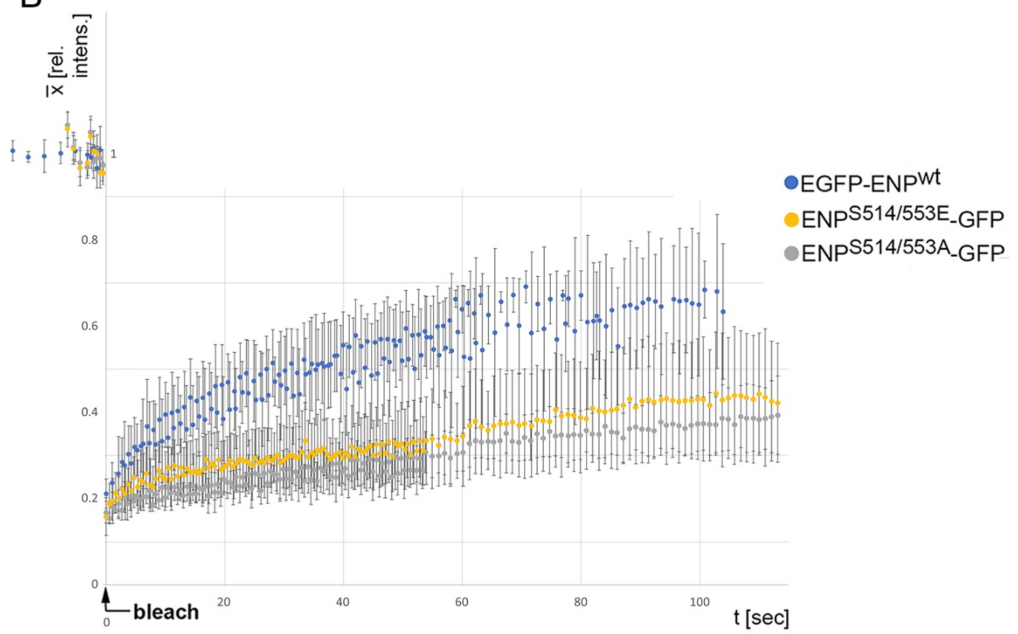

S11 Fig: FRAP analyses of ENP constructs

Shown is the recovery of ENP full length and double mutant ENPS514A/S553A and ENPS514E/S553E constructs applying bleaching spots of 40µm^2 diameter (A) and 60µm^2 (B). Note, that in particular the recovery dynamics of all constructs carrying the GFP6 at the C-terminus lay close together in all experiments. The recovery of the ENP full length construct carrying EGFP at the N-terminus was closer to the other constructs in the 40µm^2 bleach spot experiment than in the experiment with 20µm^2 (Fig. 3) and

60µm^2 diameter bleach spot. This might be due to different growth behavior of the sibling line from which the seedlings originated. Note, that the relative intensities are equal in the experiments, only the scale is shifted vertically for the intensities in B.
