# Supplementary material for: Distinct domains of ENHANCER OF PINOID hold information for its polarization required for auxin-mediated cotyledon and flower development in Arabidopsis
Source: PLoS Genet. 2025 Jun 23;21(6):e1011217. doi: 10.1371/journal.pgen.1011217 (PMC12201645; doi:10.1371/journal.pgen.1011217)
Supplement: S12 Fig — (PDF) [file pgen.1011217.s014.pdf]

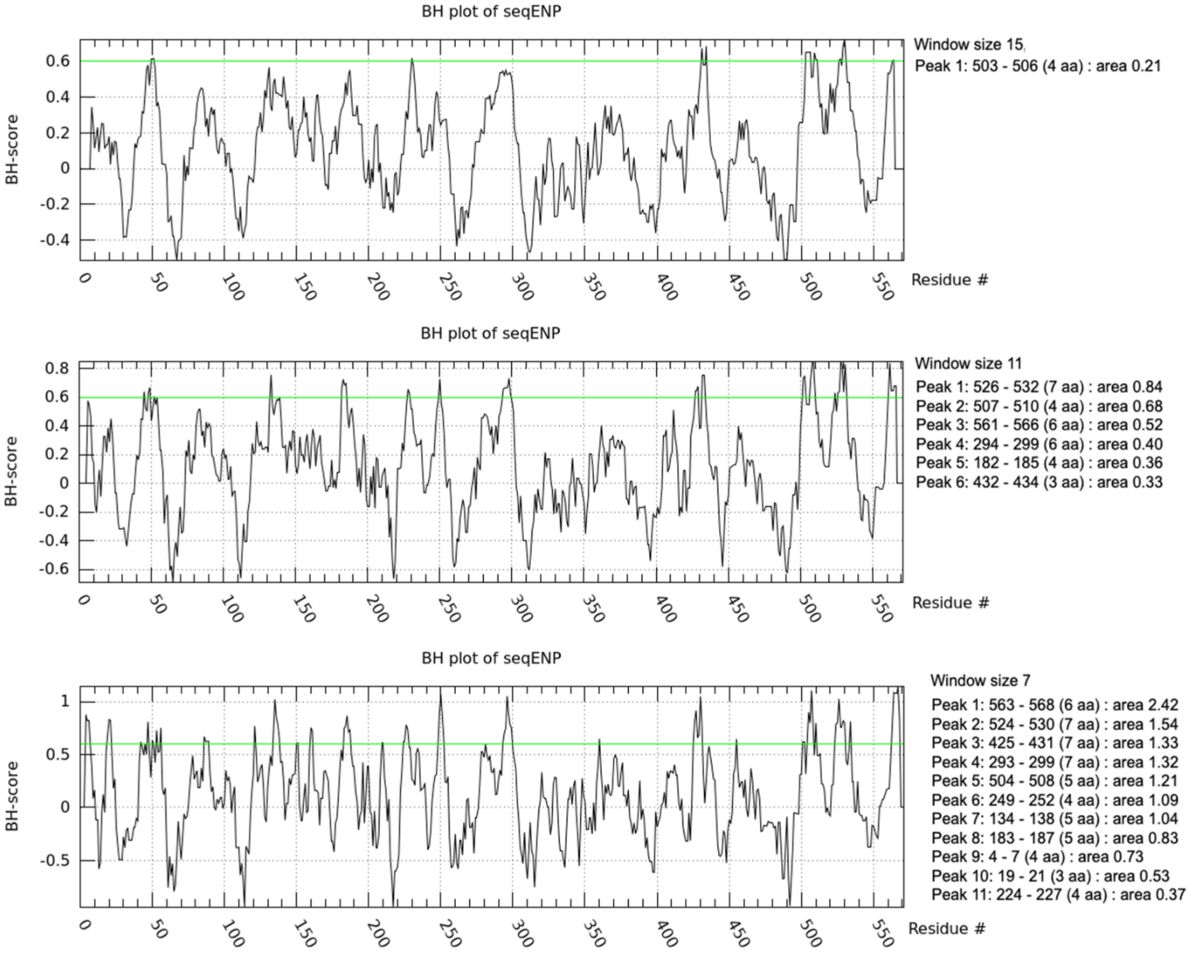

S12 Fig: Basic and Hydrophobic plot scores of ENP

Shown are three different Basic Hydrophobic plots [1] of ENP. With reduced window size, short amino acid sequence segments (from 3aas to 7 aas), along the whole ENP sequence cross the critical BH-score (green line) including the BTB/POZ, linker, NPH3-similar domain and the C-terminal IDR. Brzeska et al. [1] explored window sizes of 19aas and 11aas to detect potential PM-associated aa segments in various genes.

Literature

1. Brzeska H, Guag J, Remmert K, Chacko S, Korn ED (2010) An Experimentally Based Computer Search Identifies Unstructured Membrane-binding Sites in Proteins *APPLICATION TO CLASS I MYOSINS, PAKS, AND CARMIL.* J Biol Chem 285: 5738-5747.
