# Supplementary material for: Distinct domains of ENHANCER OF PINOID hold information for its polarization required for auxin-mediated cotyledon and flower development in Arabidopsis
Source: PLoS Genet. 2025 Jun 23;21(6):e1011217. doi: 10.1371/journal.pgen.1011217 (PMC12201645; doi:10.1371/journal.pgen.1011217)
Supplement: S2 Data — (DOCX) [file pgen.1011217.s016.docx]

**S2 Dataset: Table of ENP function restoration/“rescue“ experiments**

| Genotype of seed-lings, resistance | Comment | No. | Bracts/leaf structures | Flower structures | | | | Seeds/Comments |
| --- | --- | --- | --- | --- | --- | --- | --- | --- |
| *pid-15/pid-15* | Point mutant, not transgenic |  |  | Sepals | Petals | Stamen | Gynoecia |  |
|  |  | 1 | + | + | + | + | + | 100-200/wt |
|  |  | 2 | + | + | + | + | + | 100-200/wt |
|  |  | 3 | + | + | + | + | + | 31/wt |
|  |  | 4 | + | + | + | + | + | > 200/wt |
|  |  | 5 | + | + | + | + | + | > 200/wt |
|  |  | 6 | + | + | + | + | + | 51/wt |
|  |  | 7 | + | + | + | + | + | 100-200/wt |
|  |  | 8 | + | + | + | + | + | 100-200/wt |
|  |  | 9 | + | + | + | + | + | 38/wt + 1 lethal |
|  |  | 10 | + | + | + | + | + | 100-200/wt + 10 lethal |
|  |  | 11 | + | + | + | + | + | 34/wt + 7 lethal |
|  |  | 12 | + | + | + | + | + | 19/wt + 5 lethal |
|  |  | 13 | + | + | + | + | + | 49/wt +1 lethal |
|  |  | 14 | + | + | + | + | + | > 200/wt |
|  |  | 15 | + | + | + | + | + | > 200/wt |
|  |  | 16 | + | + | + | + | + | > 200/wt |
|  |  | 17 | + | + | + | + | + | > 200/wt |
|  |  | 18 | + | + | + | + | + | > 200/wt |
|  |  | 19 | + | + | + | + | + | > 200/wt |
|  |  | 20 | + | + | + | + | + | > 200/wt |
|  |  | 21 | + | + | + | + | + | > 200/wt |
| ∑ |  | 21* | 100% | 100% | 100% | 100% | 100% | 100% |
|  |  |  |  |  |  |  |  |  |

wt: wild-type

lethal: dark, likely non-viable seeds, *laterne* morphology often recognizable

*: no. of seedlings explicitly analysed for this Table, many more analysed during the study with similar result

| Genotype of seed-lings, resistance | Comment | No. | Bracts/leaf structures | Flower structures^1^ | | | | Seeds/Comments |
| --- | --- | --- | --- | --- | --- | --- | --- | --- |
| *enp pid/enp pid (laterne)** | Point mutant, not transgenic |  |  | Sepals | Petals | Stamen | Gynoecia |  |
|  |  | 1-20 | - | - | - | - | - | 0 |
| ∑ |  | 20* | 0% | 0% | 0% | 0% | 0% | 0% |
|  |  |  |  |  |  |  |  |  |

*: no. of seedlings explicitly analysed for this Table, many more analysed during the study with similar result

| Genotype of seed-lings, resistance | Comment | No. | Bracts/leaf structures | Flower structures^1^ | | | | Seeds/Comments |
| --- | --- | --- | --- | --- | --- | --- | --- | --- |
| *enp pid/enp pid (laterne) & ENP full length = EGFP-ENP, Basta^res.^* | Transgenic plant A descendants |  |  | Sepals | Petals | Stamen | Gynoecia |  |
|  |  | 1 | + | + | + | + | + | 88/lat + 1 lethal |
|  |  | 2 | + | + | + | + | + | 70/lat |
|  |  | 3 | + | + | + | + | + | 12/lat + 1 lethal |
|  |  | 4 | + | + | + | + | + | 33/lat + 2 lethal |
|  |  | 5 | + | + | + | + | + | 120/lat + 4 lethal |
|  |  | 6 | + | + | + | + | + | 29/lat |
|  |  | 7 | + | + | + | + | + | 8/lat |
|  |  | 8 | + | + | + | + | + | 41/lat + 2 lethal |
|  |  | 9 | + | + | + | + | + | 339/lat + 1 lethal |
|  |  | 10 | + | + | + | + | + | 109/lat + 8 lethal |
|  |  | 11 | + | + | + | + | + | 23/lat + 2 lethal |
|  |  | 12 | + | + | + | + | + | 31/lat |
|  |  | 13 | + | + | + | + | + | 305/lat + 3 lethal |
|  |  | 14 | + | + | + | + | + | 34/lat + 5 lethal |
|  |  | 15 | + | + | + | + | + | 50/lat + 3 lethal |
|  |  | 16 | + | + | + | + | + | 247/lat + 9 lethal |
|  |  | 17 | + | + | + | + | + | 169/lat + 2 lethal |
| ∑ |  | 17 | 100% | 100% | 100% | 100% | 100% |  |
|  |  |  |  |  |  |  |  |  |
| *enp pid/enp pid (laterne) & ENP full length = ENP-GFP6, Hygrom.^res^* | Transgenic plant B descendants |  |  | Sepals | Petals | Stamen | Gynoecia |  |
|  |  | 1 | + | + | + | + | + | 335/lat + 15 lethal |
|  |  | 2 | + | + | + | + | + | 103/lat + 10 lethal |
|  |  | 3 | + | + | + | + | + | 140/lat + 13 lethal |
|  |  | 4 | + | + | + | + | - | 0 |
|  |  | 5 | + | + | + | + | + | 2/lat + 1 lethal |
|  |  | 6 | + | + | + | + | + | 95/lat + 9 lethal |
|  |  | 7 | + | + | + | + | + | 44/lat + 3 lethal |
|  |  | 8 | + | + | + | + | + | 155/lat + 7 lethal |
|  |  | 9 | + | + | + | + | + | 75/lat + 3 lethal |
|  |  | 10 | + | + | + | + | + | 1/lat + 1 lethal |
|  |  | 11 | + | + | + | + | - | 0 |
|  |  | 12 | + | + | + | + | + | 87/lat + 7 lethal |
|  |  | 13 | + | + | + | + | + | 0 |
|  |  | 14 | + | + | + | + | + | 108/lat + 3 lethal |
|  |  | 15 | + | + | + | + | + | 75/lat + 5 lethal |
|  |  | 16 | + | + | + | + | + | 100/lat + 8 lethal |
|  |  | 17 | + | + | + | + | + | 1/lat |
| ∑ |  | 17 | 100% | 100% | 100% | 100% | 88% |  |
|  |  |  |  |  |  |  |  |  |

lat: *laterne*:= *enp pid/enp pid*

lethal: dark, likely non-viable seeds, *laterne* morphology often recognizable

| Genotype of seed-lings, resistance | Comment | No. | Bracts/leaf structures | Flower structures^1^ | | | | Seeds/Comments |
| --- | --- | --- | --- | --- | --- | --- | --- | --- |
| *enp pid/enp pid* (laterne)&ENP∆Cterm-GFP6, Hygrom.^res^* | Transgenic plant 1 descendants |  |  | Sepals | Petals | Stamen | Gynoecia |  |
|  |  | 1-11 | - | - | - | - | - | 0 |
| ∑ |  | 11 | 0% | 0% | 0% | 0% | 0% | 0% |
|  |  |  |  |  |  |  |  |  |
| *enp pid/enp pid (laterne)&ENP∆Cterm-GFP6, Hygrom.^res^* | Transgenic plant 2 descendants |  |  | Sepals | Petals | Stamen | Gynoecia |  |
|  |  | 1-58 | - | - | - | - | - | 0 |
| ∑ |  | 58 | 0% | 0% | 0% | 0% | 0% | 0% |
|  |  |  |  |  |  |  |  |  |

| Genotype of seed-lings, resistance | Comment | No. | Bracts/leaf structures | Flower structures^1^ | | | | Seeds/Comments |
| --- | --- | --- | --- | --- | --- | --- | --- | --- |
| *enp pid/enp pid (laterne)&EGFP-ENP∆Nterm,Basta^res.^* | Transgenic plant 1 descendants |  |  | Sepals | Petals | Stamen | Gynoecia |  |
|  |  | 1-33 | - | - | - | - | - | 0 |
| ∑ |  | 33 | 0% | 0% | 0% | 0% | 0% | 0% |
|  |  |  |  |  |  |  |  |  |

| Genotype of seed-lings, resistance | Comment | No. | Bracts/leaf structures | Flower structures^1^ | | | | Seeds/Comments |
| --- | --- | --- | --- | --- | --- | --- | --- | --- |
| *enp pid/enp pid (laterne)&ENP∆NPH3_3-GFP6, Hygrom.^res^* | Transgenic plant 1 descendants |  |  | Sepals | Petals | Stamen | Gynoecia |  |
|  |  | 1-58 | - | - | - | - | - | 0 |
| ∑ |  | 58 | 0% | 0% | 0% | 0% | 0% | 0% |
|  |  |  |  |  |  |  |  |  |

| Genotype of seed-lings, resistance | Comment | No. | Bracts/leaf structures | Flower structures^1^ | | | | Seeds/Comments |
| --- | --- | --- | --- | --- | --- | --- | --- | --- |
| *enp pid/enp pid (laterne) & ENPCtermOnly-GFP6, Hygrom.^res^* | Transgenic plant 1 descendants |  |  | Sepals | Petals | Stamen | Gynoecia |  |
|  |  | 1-25 | - | - | - | - | - | 0 |
| ∑ |  | 25 | 0% | 0% | 0% | 0% | 0% | 0% |
|  |  |  |  |  |  |  |  |  |

| Genotype of seed-lings, resistance | Comment | No. | Bracts/leaf structures | Flower structures^1^ | | | | Seeds/Comments |
| --- | --- | --- | --- | --- | --- | --- | --- | --- |
| *enp pid/enp pid (laterne) & ENP-S514A-GFP6, Hygrom.^res^* | Transgenic plant 1 descendants |  |  | Sepals | Petals | Stamen | Gynoecia |  |
|  |  | 1-9 | - | - | - | - | - | 0 |
| ∑ |  | 9 | 0% | 0% | 0% | 0% | 0% | 0% |
|  |  |  |  |  |  |  |  |  |
| *enp pid/enp pid (laterne) & ENP-S514A-GFP6, Hygrom.^res^* | Transgenic plant 2 descendants |  |  | Sepals | Petals | Stamen | Gynoecia |  |
|  |  | 1-40 | - | - | - | - | - | 0 |
| ∑ |  | 40 | 0% | 0% | 0% | 0% | 0% | 0% |
|  |  |  |  |  |  |  |  |  |
| *enp pid/enp pid (laterne) & ENP-S514A-GFP6, Hygrom.^res^* | Transgenic plant 3 descendants |  |  | Sepals | Petals | Stamen | Gynoecia |  |
|  |  | 1-15 | - | - | - | - | - | 0 |
| ∑ |  | 15 | 0% | 0% | 0% | 0% | 0% | 0% |
|  |  |  |  |  |  |  |  |  |

| Genotype of seed-lings, resistance | Comment | No. | Bracts/leaf structures | Flower structures^1^ | | | | Seeds/Comments |
| --- | --- | --- | --- | --- | --- | --- | --- | --- |
| *enp pid/enp pid (laterne) & ENP-S553A-GFP6, Hygrom.^res^* | Transgenic plant 1 descendants |  |  | Sepals | Petals | Stamen | Gynoecia |  |
|  |  | 1-22 | - | - | - | - | - | 0 |
| ∑ |  | 22 | 0% | 0% | 0% | 0% | 0% | 0% |
|  |  |  |  |  |  |  |  |  |
| *enp pid/enp pid (laterne) & ENP-S553A-GFP6, Hygrom.^res^* | Transgenic plant 2 descendants |  |  | Sepals | Petals | Stamen | Gynoecia |  |
|  |  | 1-21 | - | - | - | - | - | 0 |
| ∑ |  | 21 | 0% | 0% | 0% | 0% | 0% | 0% |
|  |  |  |  |  |  |  |  |  |
| *enp pid/enp pid (laterne) & ENP-S553A-GFP6, Hygrom.^res^* | Transgenic plant 3 descendants |  |  | Sepals | Petals | Stamen | Gynoecia |  |
|  |  | 1-10 | - | - | - | - | - | 0 |
| ∑ |  | 10 | 0% | 0% | 0% | 0% | 0% | 0% |
|  |  |  |  |  |  |  |  |  |
| *enp pid/enp pid (laterne) & ENP-S553A-GFP6, Hygrom.^res^* | Transgenic plant 4 descendants |  |  | Sepals | Petals | Stamen | Gynoecia |  |
|  |  | 1-7 | - | - | - | - | - | 0 |
| ∑ |  | 7 | 0% | 0% | 0% | 0% | 0% | 0% |
|  |  |  |  |  |  |  |  |  |

| Genotype of seed-lings, resistance | Comment | No. | Bracts/leaf structures | Flower structures^1^ | | | | Seeds/Comments |
| --- | --- | --- | --- | --- | --- | --- | --- | --- |
| *enp pid/enp pid (laterne) & ENP-S514E-GFP6, Hygrom.^res^* | Transgenic plant 1 descendants |  |  | Sepals | Petals | Stamen | Gynoecia |  |
|  |  | 1 | + | + | + | + | + | 0 |
|  |  | 2 | + | + | + | + | + | 0 |
|  |  | 3 | + | + | + | + | + | 0 |
|  |  | 4 | + | + | + | + | + | 0 |
|  |  | 5 | + | + | + | + | + | 58 lat |
|  |  | 6 | + | + | + | + | + | 36 lat + 3 lethal |
|  |  | 7 | + | + | + | + | + | 57 lat + 3 lethal |
|  |  | 8 | + | + | + | + | + | 49 lat |
|  |  | 9 | + | + | + | + | + | 0 |
| ∑ |  | 9 | 100% | 100% | 100% | 100% | 100% | 44.4% |
|  |  |  |  |  |  |  |  |  |
| *enp pid/enp pid (laterne) & ENP-S514E-GFP6, Hygrom.^res^* | Transgenic plant 2 descendants |  |  | Sepals | Petals | Stamen | Gynoecia |  |
|  |  | 1 | + | + | + | + | + | 28 lat |
|  |  | 2 | + | + | + | + | + | 0 |
|  |  | 3 | + | + | + | + | + | 0 |
|  |  | 4 | + | + | + | + | + | 0 |
|  |  | 5 | + | + | + | + | + | 0 |
|  |  | 6 | + | + | + | + | + | 0 |
|  |  | 7 | + | + | + | + | + | 0 |
|  |  | 8 | + | + | + | + | + | 0 |
|  |  | 9 | + | + | + | + | + | 3 lat |
|  |  | 10 | + | + | + | + | + | 1 lat |
|  |  | 11 | + | + | + | + | + | 0 |
|  |  | 12 | + | + | + | + | + | 0 |
|  |  | 13 | + | + | + | + | + | 35 lat + 1 lethal |
|  |  | 14 | + | + | + | + | + | 0 |
|  |  | 15 | + | + | + | + | + | 1 lat |
|  |  | 16 | + | + | + | + | + | 0 |
| ∑ |  | 16 | 100% | 100% | 100% | 100% | 100% | 25% |
|  |  |  |  |  |  |  |  |  |
| *enp pid/enp pid (laterne) & ENP-S514E-GFP6, Hygrom.^res^* | Transgenic plant 3 descendants |  |  | Sepals | Petals | Stamen | Gynoecia |  |
|  |  | 1 | + | + | + | + | + | 11 lat |
|  |  | 2 | + | + | + | + | + | 1 lat |
|  |  | 3 | + | + | + | + | + | 0 |
|  |  | 4 | + | + | + | + | + | 0 |
|  |  | 5 | + | + | + | + | + | 2 lat + 1lethal |
|  |  | 6 | + | + | + | + | + | 1 lat |
|  |  | 7 | + | + | + | + | + | 0 |
|  |  | 8 | + | + | + | + | - | 0 |
|  |  | 9 | + | + | + | + | - | 0 |
|  |  | 10 | + | + | + | + | - | 0 |
|  |  | 11 | + | - | - | - | - | 0 |
|  |  | 12 | + | + | + | + | - | 0 |
|  |  | 13 | + | + | + | + | - | 0 |
|  |  | 14 | + | + | + | - | - | 0 |
|  |  | 15 | + | - | - | - | - | 0 |
|  |  | 16 | + | + | + | + | + | 0 |
| ∑ |  | 16 | 100% | 87.5% | 87.5% | 81.25% | 50% | 25% |
|  |  |  |  |  |  |  |  |  |
|  |  |  |  |  |  |  |  |  |
| *enp pid/enp pid (laterne) & ENP-S514E-GFP6, Hygrom.^res^* | Transgenic plant 4 descendants |  |  | Sepals | Petals | Stamen | Gynoecia |  |
|  |  | 1 | + | + | + | + | + | 0 |
|  |  | 2 | + | + | + | + | + | 0 |
|  |  | 3 | + | + | + | + | + | 6 lats |
|  |  | 4 | + | + | + | - | - | 0 |
|  |  | 5 | + | + | + | + | + | 0 |
|  |  | 6 | + | + | + | + | + | 0 |
|  |  | 7 | + | + | + | + | + | 0 |
|  |  | 8 | + | + | + | + | + | 0 |
|  |  | 9 | + | + | + | + | + | 0 |
|  |  | 10 | + | + | + | + | + | 5 lats |
|  |  | 11 | + | + | + | + | + | 0 |
|  |  | 12 | + | + | + | + | + | 0 |
|  |  | 13 | + | + | + | + | + | 2 lats |
|  |  | 14 | + | + | + | + | + | 0 |
|  |  | 15 | + | + | + | + | + | 0 |
|  |  | 16 | + | + | + | + | + | 0 |
|  |  | 17 | + | + | - | + | - | 0 |
|  |  | 18 | + | + | + | + | + | 0 |
|  |  | 19 | + | + | + | + | + | 0 |
|  |  | 20 | + | + | + | + | + | 0 |
|  |  | 21 | + | - | - | + | - | 0 |
|  |  | 22 | + | + | + | - | - | 0 |
|  |  | 23 | + | + | + | - | - | 0 |
|  |  | 24 | + | + | + | + | + | 20 lat +3 lethal |
|  |  | 25 | + | + | + | + | - | 0 |
|  |  | 26 | + | + | + | + | + | 0 |
|  |  | 27 | + | + | + | + | - | 0 |
|  |  | 28 | + | + | + | + | + | 0 |
|  |  | 29 | + | + | + | + | + | 0 |
|  |  | 30 | + | + | + | + | + | 0 |
|  |  | 31 | + | + | + | + | + | 0 |
|  |  | 32 | + | + | + | + | + | 33 lats |
|  |  | 33 | + | + | + | + | - | 0 |
|  |  | 34 | - | - | - | - | - | 0 |
|  |  | 35 | + | + | + | + | + | 6 lats |
|  |  | 36 | + | + | + | + | - | 0 |
|  |  | 37 | + | + | + | + | - | 0 |
|  |  | 38 | + | + | + | + | - | 0 |
|  |  | 39 | + | + | + | + | - | 0 |
|  |  | 40 | + | + | + | + | - | 0 |
|  |  | 41 | + | + | + | + | - | 0 |
|  |  | 42 | + | + | + | + | - | 0 |
|  |  | 43 | + | + | + | + | - | 0 |
|  |  | 44 | + | + | + | + | + | 7 lat |
|  |  | 45 | + | + | + | + | + | 37 lat |
|  |  | 46 | + | + | + | + | + | 3 lat |
|  |  | 47 | + | + | + | + | + | 0 |
|  |  | 48 | + | + | + | + | + | 0 |
|  |  | 49 | + | + | + | + | + | 5 lat |
|  |  | 50 | + | + | + | + | + | 0 |
|  |  | 51 | + | + | + | + | + | 20 lat |
|  |  | 52 | + | + | + | + | + | 10 lat |
|  |  | 53 | + | + | + | + | + | 1 lat |
|  |  | 54 | + | + | + | + | + | 0 |
|  |  | 55 | + | + | + | + | + | 49 lat + 2 lethal |
|  |  | 56 | + | + | + | + | - | 0 |
|  |  | 57 | + | + | + | + | + | 5 lat |
|  |  | 58 | + | + | + | + | + | 0 |
|  |  | 59 | + | + | + | + | + | 32 lat + 1lethal |
|  |  | 60 | + | + | + | + | + | 2 lat |
|  |  | 61 | + | + | + | + | + | 23 lat + 1lethal |
|  |  | 62 | + | + | + | + | + | 30 lat + 1lethal |
|  |  | 63 | + | + | + | + | - | 0 |
|  |  | 64 | + | + | + | + | + | 25 lat + 1 lethal |
|  |  | 65 | + | + | + | + | + | 27 lat |
| ∑ |  | 65 | 98.5% | 96.9% | 95.3% | 93.8% | 70.8% | 32.3% |
|  |  |  |  |  |  |  |  |  |

lat: *laterne*:= *enp pid/enp pid*

lethal: dark, likely non-viable seeds, *laterne* morphology often recognizable

| Genotype of seed-lings, resistance | Comment | No. | Bracts/leaf structures | Flower structures^1^ | | | | Seeds/Comments |
| --- | --- | --- | --- | --- | --- | --- | --- | --- |
| *enp pid/enp pid (laterne) & ENP-S553E-GFP6, Hygrom.^res^* | Transgenic plant 1 descendants |  |  | Sepals | Petals | Stamen | Gynoecia |  |
|  |  | 1 | + | - | + | - | - | 0 |
|  |  | 2 | + | - | + | - | - | 0 |
|  |  | 3 | + | - | + | - | - | 0 |
|  |  | 4 | + | - | + | - | - | 0 |
|  |  | 5 | + | + | + | - | - | 0 |
|  |  | 6 | - | - | - | - | + | 0 |
|  |  | 7 | - | - | + | - | + | 0 |
|  |  | 8 | + | + | + | + | - | 0 |
|  |  | 9 | + | + | - | - | - | 0 |
|  |  | 10 | + | - | - | - | - | 0 |
|  |  | 11 | + | + | + | - | - | 0 |
|  |  | 12 | + | - | - | - | - | 0 |
|  |  | 13 | + | - | - | - | - | 0 |
| ∑ |  | 13 | 84.6% | 30.8% | 61.5% | 7.7% | 15.4% | 0% |
|  |  |  |  |  |  |  |  |  |
| *enp pid/enp pid (laterne) & ENP-S553E-GFP6, Hygrom.^res^* | Transgenic plant 2 descendants |  |  | Sepals | Petals | Stamen | Gynoecia |  |
|  |  | 1 | + | - | - | - | - | 0 |
|  |  | 2 | + | + | + | + | + | 0 |
|  |  | 3 | + | - | - | - | - | 0 |
|  |  | 4 | + | - | - | - | - | 0 |
|  |  | 5 | + | - | - | - | - | 0 |
|  |  | 6 | + | - | - | - | - | 0 |
|  |  | 7 | + | - | - | - | - | 0 |
|  |  | 8 | + | - | - | - | - | 0 |
|  |  | 9 | + | + | + | - | - | 0 |
|  |  | 10 | + | - | - | - | - | 0 |
|  |  | 11 | + | - | - | - | - | 0 |
|  |  | 12 | + | - | - | - | - | 0 |
|  |  | 13 | + | - | - | - | - | 0 |
|  |  | 14 | + | - | - | - | - | 0 |
|  |  | 15 | + | + | + | - | - | 0 |
|  |  | 16 | + | - | - | - | - | 0 |
|  |  | 17 | + | - | - | - | - | 0 |
|  |  | 18 | + | + | + | + | - | 0 |
|  |  | 19 | + | - | - | - | - | 0 |
|  |  | 20 | + | + | + | - | - | 0 |
|  |  | 21 | + | + | - | + | - | 0 |
|  |  | 22 | + | + | - | - | - | 0 |
|  |  | 23 | + | + | - | - | - | 0 |
|  |  | 24 | + | - | - | - | - | 0 |
|  |  | 25 | + | - | - | - | - | 0 |
|  |  | 26 | + | - | + | - | - | 0 |
| ∑ |  | 26 | 100% | 30.8% | 23.1% | 11.5% | 3.8% | 0% |
|  |  |  |  |  |  |  |  |  |
| *enp pid/enp pid (laterne) & ENP-S553E-GFP6, Hygrom.^res^* | Transgenic plant 3 descendants |  |  | Sepals | Petals | Stamen | Gynoecia |  |
|  |  | 1 | - | - | - | - | - | 0 |
|  |  | 2 | - | - | - | - | - | 0 |
|  |  | 3 | + | - | - | - | - | 0 |
|  |  | 4 | + | - | - | - | - | 0 |
|  |  | 5 | - | - | - | - | - | 0 |
|  |  | 6 | - | - | - | - | - | 0 |
|  |  | 7 | - | - | - | - | - | 0 |
|  |  | 8 | - | - | - | - | - | 0 |
|  |  | 9 | - | - | - | - | - | 0 |
|  |  | 10 | - | - | - | - | + | 0 |
|  |  | 11 | + | - | - | + | + | 0 |
|  |  | 12 | - | - | - | + | - | 0 |
| ∑ |  | 12 | 15% | 0% | 0% | 16.7% | 16.7% | 0% |
|  |  |  |  |  |  |  |  |  |
| *enp pid/enp pid (laterne) & ENP-S553E-GFP6, Hygrom.^res^* | Transgenic plant 4 descendants |  |  | Sepals | Petals | Stamen | Gynoecia |  |
|  |  | 1 | + | - | - | - | - | 0 |
|  |  | 2 | + | - | - | - | - | 0 |
|  |  | 3 | + | - | + | - | - | 0 |
|  |  | 4 | - | - | - | - | - | 0 |
|  |  | 5 | - | + | + | - | - | 0 |
|  |  | 6 | + | - | + | - | - | 0 |
|  |  | 7 | + | - | - | - | - | 0 |
|  |  | 8 | + | - | - | - | - | 0 |
|  |  | 9 | - | - | - | - | - | 0 |
|  |  | 10 | + | - | + | - | - | 0 |
|  |  | 11 | + | - | - | - | - | 0 |
|  |  | 12 |  |  |  |  |  |  |
| ∑ |  | 12 | 72.7% | 9.1% | 36.4% | 0% | 0% | 0% |
|  |  |  |  |  |  |  |  |  |

| Genotype of seed-lings, resistance | Comment | No. | Bracts/leaf structures | Flower structures^1^ | | | | Seeds/Comments |
| --- | --- | --- | --- | --- | --- | --- | --- | --- |
| *enp pid/enp pid (laterne) & ENP-S514A/S553A-GFP6, Hygrom.^res^* | Transgenic plant 1 descendants |  |  | Sepals | Petals | Stamen | Gynoecia |  |
|  |  | 1 | + | - | - | - | - | 0 |
|  |  | 2 | + | - | + | - | - | 0 |
|  |  | 3 | + | - | - | - | - | 0 |
|  |  | 4 | + | - | - | - | - | 0 |
|  |  | 5 | - | - | - | - | - | 0 |
|  |  | 6 | + | - | - | - | - | 0 |
|  |  | 7 | + | - | - | - | - | 0 |
|  |  | 8 | + | - | + | - | - | 0 |
|  |  | 9 | + | - | - | - | - | 0 |
|  |  | 10 | - | - | - | - | + | 0 |
|  |  | 11 | + | - | - | - | - | 0 |
|  |  | 12 | + | - | - | - | - | 0 |
|  |  | 13 | + | - | + | - | - | 0 |
|  |  | 14 | + | - | + | - | - | 0 |
|  |  | 15 | + | - | + | - | - | 0 |
|  |  | 16 | + | - | + | - | - | 0 |
|  |  | 17 | + | - | - | - | - | 0 |
|  |  | 18 | + | - | - | - | - | 0 |
|  |  | 19 | + | - | - | - | - | 0 |
|  |  | 20 | + | - | - | - | - | 0 |
|  |  | 21 | + | - | + | - | - | 0 |
|  |  | 22 | + | - | - | - | - | 0 |
|  |  | 23 | + | + | + | + | - | 0 |
| ∑ |  | 23 | 91.3% | 4.3% | 34.8% | 4.3% | 4.3% | 0% |
|  |  |  |  |  |  |  |  |  |
| *enp pid/enp pid (laterne) & ENP-S514A/S553A-GFP6, Hygrom.^res^* | Transgenic plant 2 descendants |  |  | Sepals | Petals | Stamen | Gynoecia |  |
|  |  | 1 | + | + | - | - | - | 0 |
|  |  | 2 | + | + | + | + | + | 0 |
|  |  | 3 | + | + | + | + | - | 0 |
|  |  | 4 | + | + | + | + | + | 0 |
|  |  | 5 | + | + | + | + | + | 0 |
|  |  | 6 | + | + | + | + | + | 0 |
|  |  | 7 | + | + | + | + | + | 0 |
|  |  | 8 | + | + | + | + | + | 2 lat |
|  |  | 9 | + | + | + | + | + | 2 lat |
|  |  | 10 | + | + | + | + | - | 0 |
|  |  | 11 | + | + | + | + | - | 0 |
|  |  | 12 | + | + | + | + | - | 0 |
|  |  | 13 | + | + | + | + | + | 6 lat |
|  |  | 14 | + | + | + | + | + | 4 lat |
|  |  | 15 | + | + | + | + | + | 16 lat |
|  |  | 16 | + | + | + | + | - | 0 |
|  |  | 17 | + | + | + | + | + | 0 |
|  |  | 18 | + | + | + | + | + | 0 |
|  |  | 19 | + | + | + | + | + | 3 lat + 1 lethal |
|  |  | 20 | + | + | + | + | + | 54 lat + 1 lethal |
|  |  | 21 | + | + | + | + | + | 0 |
|  |  | 22 | + | + | + | + | + | 0 |
|  |  | 23 | + | + | + | + | - | 0 |
|  |  | 24 | + | + | + | + | + | 1 lat |
|  |  | 25 | + | + | + | + | + | 22 lat + 1 lethal |
|  |  | 26 | + | + | + | + | + | 1lat |
|  |  | 27 | + | + | + | + | + | 10 lat |
|  |  | 28 | + | + | + | + | + | 0 |
|  |  | 29 | + | + | + | + | + | 15 lat |
|  |  | 30 | + | + | + | + | - | 0 |
|  |  | 31 | + | + | + | + | - | 0 |
|  |  | 32 | + | + | + | + | + | 5 lat |
|  |  | 33 | + | + | + | + | + | 0 |
|  |  | 34 | + | + | + | + | + | 0 |
|  |  | 35 | + | + | + | + | - | 0 |
|  |  | 36 | + | + | + | + | - | 0 |
|  |  | 37 | + | + | + | + | + | 0 |
|  |  | 38 | + | + | + | + | + | 1 lat |
|  |  | 39 | + | + | + | + | - | 0 |
|  |  | 40 | + | + | + | + | + | 4 lat |
|  |  | 41 | + | + | + | + | + | 11 lat + 1 lethal |
|  |  | 42 | + | + | + | + | + | 0 |
|  |  | 43 | + | + | + | + | - | 0 |
|  |  | 44 | + | + | + | + | + | 18 lat |
|  |  | 45 | + | + | + | + | - | 0 |
|  |  | 46 | + | + | + | + | + | 0 |
|  |  | 47 | + | + | + | + | + | 31 lat |
|  |  | 48 | + | + | + | + | - | 0 |
|  |  | 49 | + | + | + | + | + | 16 lat |
|  |  | 50 | + | + | + | + | - | 0 |
|  |  | 51 | + | + | + | + | - | 0 |
|  |  | 52 | + | + | + | + | + | 0 |
|  |  | 53 | + | + | + | + | - | 0 |
|  |  | 54 | + | + | + | + | - | 0 |
|  |  | 55 | + | + | + | + | - | 0 |
|  |  | 56 | + | + | + | + | + | 13 lat + 3 lethal |
|  |  | 57 | + | + | + | + | + | 14 lat |
|  |  | 58 | + | + | + | + | - | 0 |
|  |  | 59 | + | + | + | + | - | 0 |
|  |  | 60 | + | + | + | + | + | 20 lat + 2 lethal |
|  |  | 61 | + | + | + | + | + | 16 lat +1 lethal |
|  |  | 62 | + | + | + | + | - | 0 |
|  |  | 63 | + | + | + | + | + | 4 lat |
|  |  | 64 | + | + | + | + | + | 61 lat+3lethal |
|  |  | 65 | + | + | + | + | + | 0 |
|  |  | 66 | + | + | + | + | - | 0 |
|  |  | 67 | + | + | + | + | - | 0 |
|  |  | 68 | + | + | + | + | + | 0 |
|  |  | 69 | + | + | + | + | + | 18 lat |
| ∑ |  | 69 | 100% | 100% | 98.6% | 98.6% | 63.8% | 37.7% |
|  |  |  |  |  |  |  |  |  |
| *enp pid/enp pid (laterne) & ENP-S514A/S553A-GFP6, Hygrom.^res^* | Transgenic plant 3 descendants |  |  | Sepals | Petals | Stamen | Gynoecia |  |
|  |  | 1 | + | + | + | + | + | 0 |
|  |  | 2 | + | + | + | + | + | 0 |
|  |  | 3 | + | + | + | + | - | 0 |
|  |  | 4 | + | + | + | + | + | 0 |
|  |  | 5 | + | + | + | + | - | 0 |
|  |  | 6 | + | + | + | + | - | 0 |
|  |  | 7 | + | + | + | + | + | 1 lat |
|  |  | 8 | + | + | + | + | + | 15 lat + 2 lethal |
|  |  | 9 | + | + | + | + | + | 0 |
|  |  | 10 | + | + | + | + | - | 0 |
|  |  | 11 | + | + | + | + | - | 0 |
|  |  | 12 | + | + | + | + | + | 2 lat |
|  |  | 13 | + | + | + | + | + | 0 |
|  |  | 14 | + | + | + | + | - | 0 |
|  |  | 15 | + | + | + | + | - | 0 |
|  |  | 16 | + | + | + | + | - | 0 |
|  |  | 17 | + | + | + | + | - | 0 |
|  |  | 18 | + | + | + | + | - | 0 |
|  |  | 19 | + | + | + | + | - | 0 |
|  |  | 20 | + | + | + | + | - | 0 |
|  |  | 21 | + | + | + | + | + | 1 lat |
|  |  | 22 | + | + | + | + | + | 1 lat |
|  |  | 23 | + | + | + | + | + | 0 |
|  |  | 24 | + | + | + | + | + | 1 lat |
|  |  | 25 | + | + | + | + | - | 0 |
|  |  | 26 | + | + | + | + | - | 0 |
|  |  | 27 | + | + | + | + | + | 0 |
|  |  | 28 | + | + | + | + | + | 20 lat |
|  |  | 29 | + | + | + | + | + | 1 lat + 1 lethal |
|  |  | 30 | + | + | + | + | + | 0 |
|  |  | 31 | + | + | + | + | + | 10 lat + 1 lethal |
|  |  | 32 | + | + | + | + | + | 16 lat + 1 lethal |
|  |  | 33 | + | + | + | + | - | 0 |
|  |  | 34 | + | + | + | + | - | 0 |
|  |  | 35 | + | + | + | + | + | 15 lat +1 lethal |
|  |  | 36 | + | + | + | - | + | 0 |
|  |  | 37 | + | + | + | - | + | 0 |
|  |  | 38 | + | + | + | + | + | 0 |
|  |  | 39 | + | + | + | + | - | 0 |
|  |  | 40 | + | + | + | + | - | 0 |
| ∑ |  | 40 | 100% | 100% | 100% | 95% | 55% | 27.5% |
|  |  |  |  |  |  |  |  |  |

lat: *laterne*:= *enp pid/enp pid*

lethal: dark, likely non-viable seeds, *laterne* morphology often recognizable

| Genotype of seed-lings, resistance | Comment | No. | Bracts/leaf structures | Flower structures^1^ | | | | Seeds/Comments |
| --- | --- | --- | --- | --- | --- | --- | --- | --- |
| *enp pid/enp pid (laterne) & ENP-S514E/S553E-GFP6, Hygrom.^res^* | Transgenic plant 1 descendants |  |  | Sepals | Petals | Stamen | Gynoecia |  |
|  |  | 1 | + | + | + | + | - | 0 |
|  |  | 2 | + | + | + | + | - | 0 |
|  |  | 3 | + | + | + | + | + | 5 lat |
|  |  | 4 | + | + | + | + | + | 0 |
|  |  | 5 | + | + | + | + | - | 0 |
|  |  | 6 | + | + | + | + | - | 0 |
|  |  | 7 | + | - | + | + | - | 0 |
|  |  | 8 | + | - | + | + | - | 0 |
|  |  | 9 | + | - | - | - | - | 0 |
|  |  | 10 | + | + | + | + | - | 0 |
|  |  | 11 | + | + | + | + | + | 2 lat |
|  |  | 12 | + | + | + | + | + | 2 lat |
|  |  | 13 | + | + | + | + | + | 1 lat |
|  |  | 14 | + | + | + | + | - | 0 |
|  |  | 15 | + | + | + | + | - | 0 |
|  |  | 16 | + | + | + | + | - | 0 |
|  |  | 17 | + | + | + | + | - | 0 |
|  |  | 18 | + | + | + | + | + | 6 lat |
|  |  | 19 | + | + | + | + | - | 0 |
|  |  | 20 | + | - | - | - | - | 0 |
|  |  | 21 | + | + | + | + | - | 0 |
|  |  | 22 | + | + | + | + | - | 0 |
|  |  | 23 | + | + | + | + | + | 8 lat |
|  |  | 24 | + | + | + | + | - | 0 |
|  |  | 25 | + | + | + | + | + | 7 lat |
|  |  | 26 | + | + | + | + | - | 0 |
|  |  | 27 | + | + | + | + | - | 0 |
|  |  | 28 | + | + | + | + | + | 4 lat |
|  |  | 29 | + | + | + | + | - | 0 |
|  |  | 30 | + | + | + | + | - | 0 |
|  |  | 31 | + | + | + | + | - | 0 |
|  |  | 32 | + | + | + | + | - | 0 |
|  |  | 33 | + | + | + | + | + | 1 lat |
|  |  | 34 | + | + | + | + | + | 34 lat |
|  |  | 35 | + | + | + | + | + | 0 |
|  |  | 36 | + | + | + | + | + | 0 |
|  |  | 37 | + | + | + | + | + | 9 lat |
|  |  | 38 | + | + | + | + | + | 1 lat |
|  |  | 39 | + | + | + | + | + | 0 |
|  |  | 40 | + | + | + | + | + | 0 |
|  |  | 41 | + | + | + | + | + | 34 lat |
|  |  | 42 | + | + | + | + | + | 37 lat + 1letal |
|  |  | 43 | + | + | + | + | - | 0 |
|  |  | 44 | + | + | + | + | - | 0 |
|  |  | 45 | + | + | + | + | + | 4 lat |
|  |  | 46 | + | + | + | + | - | 0 |
|  |  | 47 | + | + | + | + | - | 0 |
|  |  | 48 | + | + | + | + | + | 0 |
|  |  | 49 | + | + | + | + | + | 1 lat |
|  |  | 50 | + | + | + | + | + | 55 lat |
|  |  | 51 | + | + | + | + | + | 31 lat |
|  |  | 52 | + | + | + | + | + | 0 |
|  |  | 53 | + | + | + | + | + | 2 lat |
|  |  | 54 | + | + | + | + | + | 1 lat |
|  |  | 55 | + | + | + | + | - | 0 |
|  |  | 56 | + | + | + | + | - | 0 |
|  |  | 57 | + | + | + | + | + | 0 |
| ∑ |  | 57 | 100% | 93% | 96.5% | 96.5% | 49.1% | 35.1% |
|  |  |  |  |  |  |  |  |  |
| *enp pid/enp pid (laterne) & ENP-S514E/S553E-GFP6, Hygrom.^res^* | Transgenic plant 2 descendants |  |  | Sepals | Petals | Stamen | Gynoecia |  |
|  |  | 1 | + | + | + | + | + | 0 |
|  |  | 2 | + | + | + | + | + | 0 |
|  |  | 3 | + | + | + | + | + | 2 lat |
|  |  | 4 | + | + | + | + | + | 0 |
|  |  | 5 | + | + | + | + | + | 9 lat |
|  |  | 6 | + | + | + | + | + | 1 lat |
|  |  | 7 | + | + | + | + | + | 1 lat |
|  |  | 8 | + | + | + | + | + | 3 lat |
|  |  | 9 | + | + | + | + | - | 0 |
|  |  | 10 | + | + | + | + | + | 0 |
|  |  | 11 | + | + | + | + | + | 1 lat |
|  |  | 12 | + | + | + | + | + | 5 lat |
|  |  | 13 | + | + | + | + | + | 9 lat |
|  |  | 14 | + | + | + | + | + | 106 lat |
|  |  | 15 | + | + | + | + | + | 7lat |
|  |  | 16 | + | + | + | + | + | 225 lat |
|  |  | 17 | + | + | + | + | + | 6 lat |
|  |  | 18 | + | + | + | + | + | 89 lat |
|  |  | 19 | + | + | + | + | + | 14 lat |
|  |  | 20 | + | + | + | + | + | 112 lat |
|  |  | 21 | + | + | + | + | + | 157 lat |
| ∑ |  | 21 | 100% | 100% | 100% | 100% | 95.2% | 76.2% |
|  |  |  |  |  |  |  |  |  |
|  |  |  |  |  |  |  |  |  |
| *enp pid/enp pid (laterne) & ENP-S514E/S553E-GFP6, Hygrom.^res^* | Transgenic plant 3 descendants |  |  | Sepals | Petals | Stamen | Gynoecia |  |
|  |  | 1 |  | + | + | + | - | 0 |
|  |  | 2 |  | + | + | + | + | 2 lat |
|  |  | 3 |  | + | + | + | - | 0 |
|  |  | 4 |  | + | + | + | + | 0 |
|  |  | 5 |  | + | + | + | + | 1 lat |
|  |  | 6 |  | + | + | + | + | 0 |
|  |  | 7 |  | + | + | + | + | 1 lat |
|  |  | 8 |  | + | + | + | + | 1 lat |
|  |  | 9 |  | + | + | + | + | 76 lat |
|  |  | 10 |  | + | + | + | + | 1 lat |
|  |  | 11 |  | + | + | + | + | 1 lat |
|  |  | 12 |  | + | + | + | + | 1 lat |
|  |  | 13 |  | + | + | + | + | 73 lat |
|  |  | 14 |  | + | + | + | - | 0 |
|  |  | 15 |  | + | + | + | + | 15 lat |
|  |  | 16 |  | + | + | + | + | 2 lat |
|  |  | 17 |  | + | + | + | - | 0 |
|  |  | 18 |  | + | + | + | + | 5 lat |
|  |  | 19 |  | + | + | + | - | 0 |
|  |  | 20 |  | + | + | + | + | 9 lat |
|  |  | 21 |  | + | + | + | - | 0 |
| ∑ |  | 21 | not analysed | 100% | 100% | 100% | 71.4% | 66.7% |
|  |  |  |  |  |  |  |  |  |

lat: *laterne*:= *enp pid/enp pid*

lethal: dark, likely non-viable seeds, *laterne* morphology often recognizable

| Genotype of seed-lings, resistance | Comment | No. | Bracts/leaf structures | Flower structures^1^ | | | | Seeds/Comments |
| --- | --- | --- | --- | --- | --- | --- | --- | --- |
| *enp pid/enp pid (laterne) & ENP-S514E/S553A-GFP6, Hygrom.^res^* | Transgenic plant 1 descendants |  |  | Sepals | Petals | Stamen | Gynoecia |  |
|  |  | 1 | - | - | - | - | - | 0 |
|  |  | 2 | - | - | - | - | - | 0 |
|  |  | 3 | - | - | - | - | - | 0 |
|  |  | 4 | + | - | + | + | - | 0 |
|  |  | 5 | - | - | - | - | - | 0 |
|  |  | 6 | - | - | - | - | - | 0 |
|  |  | 7 | - | - | - | - | - | 0 |
|  |  | 8 | + | - | - | - | - | 0 |
|  |  | 9 | - | - | - | - | - | 0 |
|  |  | 10 | - | - | - | - | - | 0 |
|  |  | 11 | - | - | - | - | - | 0 |
|  |  | 12 | - | - | - | - | - | 0 |
|  |  | 13 | + | - | - | - | - | 0 |
|  |  | 14 | + | - | + | - | - | 0 |
|  |  | 15 | - | - | + | + | - | 0 |
|  |  | 16 | - | - | - | - | - | 0 |
|  |  | 17 | - | - | - | - | - | 0 |
|  |  | 18 | + | - | - | - | - | 0 |
|  |  | 19 | - | - | - | - | - | 0 |
| ∑ |  | 19 | 26.3% | 0% | 15.8% | 10.5% | 0% | 0% |
|  |  |  |  |  |  |  |  |  |
|  |  |  |  |  |  |  |  |  |
| *enp pid/enp pid (laterne) & ENP-S514E/S553A-GFP6, Hygrom.^res^* | Transgenic plant 2 descendants |  |  | Sepals | Petals | Stamen | Gynoecia |  |
|  |  | 1 | - | - | + | - | - | 0 |
|  |  | 2 | + | - | - | - | - | 0 |
|  |  | 3 | + | - | - | - | - | 0 |
|  |  | 4 | + | - | + | - | - | 0 |
|  |  | 5 | - | - | - | - | - | 0 |
|  |  | 6 | + | - | - | - | - | 0 |
|  |  | 7 | + | - | - | - | - | 0 |
|  |  | 8 | + | - | - | - | - | 0 |
|  |  | 9 | + | - | - | - | - | 0 |
|  |  | 10 | - | - | - | - | - | 0 |
|  |  | 11 | + | - | - | - | - | 0 |
|  |  | 12 | + | + | + | + | - | 0 |
|  |  | 13 | + | - | - | - | - | 0 |
|  |  | 14 | + | + | + | + | - | 0 |
|  |  | 15 | + | - | + | - | - | 0 |
| ∑ |  | 15 | 80% | 13.3% | 33.3% | 13.3% | 0% | 0% |
|  |  |  |  |  |  |  |  |  |

| Genotype of seed-lings, resistance | Comment | No. | Bracts/leaf structures | Flower structures^1^ | | | | Seeds/Comments |
| --- | --- | --- | --- | --- | --- | --- | --- | --- |
| *enp pid/enp pid (laterne) & ENP-S514A/S553E-GFP6, Hygrom.^res^* | Transgenic plant 1 descendants |  |  | Sepals | Petals | Stamen | Gynoecia |  |
|  |  | 1 | + | - | - | - | - | 0 |
|  |  | 2 | + | - | + | + | - | 0 |
|  |  | 3 | + | - | - | - | - | 0 |
|  |  | 4 | + | - | + | - | - | 0 |
|  |  | 5 | + | + | + | + | - | 0 |
|  |  | 6 | - | + | + | + | - | 0 |
|  |  | 7 | + | + | + | + | - | 0 |
|  |  | 8 | + | + | + | - | - | 0 |
|  |  | 9 | + | - | - | - | - | 0 |
|  |  | 10 | + | + | + | - | - | 0 |
|  |  | 11 | + | - | - | - | - | 0 |
|  |  | 12 | + | + | + | - | - | 0 |
|  |  | 13 | + | + | + | - | - | 0 |
|  |  | 14 | + | + | + | + | - | 0 |
|  |  | 15 | + | + | + | + | - | 0 |
|  |  | 16 | + | - | - | - | - | 0 |
|  |  | 17 | + | - | - | - | - | 0 |
|  |  | 18 | + | + | + | - | - | 0 |
|  |  | 19 | + | + | + | - | - | 0 |
|  |  | 20 | + | - | + | + | - | 0 |
|  |  | 21 | + | - | + | - | - | 0 |
|  |  | 22 | + | + | + | + | - | 0 |
|  |  | 23 | + | + | + | + | - | 0 |
|  |  | 24 | + | - | - | - | - | 0 |
|  |  | 25 | + | + | + | - | - | 0 |
|  |  | 26 | + | - | + | - | - | 0 |
|  |  | 27 | + | + | + | + | - | 0 |
|  |  | 28 | + | + | + | + | - | 0 |
|  |  | 29 | + | + | + | + | - | 0 |
| ∑ |  | 29 | 96.6% | 58.6% | 75.9% | 41.4% | 0% | 0% |
|  |  |  |  |  |  |  |  |  |
| *enp pid/enp pid (laterne) & ENP-S514A/S553E-GFP6, Hygrom.^res^* | Transgenic plant 2 descendants |  |  | Sepals | Petals | Stamen | Gynoecia |  |
|  |  | 1 | + | - | - | - | - | 0 |
|  |  | 2 | + | - | - | - | - | 0 |
|  |  | 3 | - | - | - | - | - | 0 |
|  |  | 4 | + | - | - | - | - | 0 |
|  |  | 5 | + | - | - | - | - | 0 |
|  |  | 6 | - | - | - | - | - | 0 |
|  |  | 7 | + | - | - | - | - | 0 |
|  |  | 8 | + | - | - | - | - | 0 |
|  |  | 9 | + | - | - | - | - | 0 |
|  |  | 10 | + | - | - | - | - | 0 |
|  |  | 11 | + | - | - | - | - | 0 |
|  |  | 12 | + | - | - | - | - | 0 |
|  |  | 13 | + | - | - | - | - | 0 |
|  |  | 14 | + | - | - | - | - | 0 |
|  |  | 15 | - | - | - | - | - | 0 |
|  |  | 16 | + | - | - | - | - | 0 |
|  |  | 17 | + | - | - | - | - | 0 |
|  |  | 18 | + | - | - | - | - | 0 |
|  |  | 19 | + | - | - | - | - | 0 |
|  |  | 20 | - | - | - | - | - | 0 |
|  |  | 21 | + | - | - | - | - | 0 |
|  |  | 22 | + | - | - | - | - | 0 |
|  |  | 23 | + | - | - | - | - | 0 |
|  |  | 24 | + | - | - | - | - | 0 |
|  |  | 25 | + | - | - | - | - | 0 |
|  |  | 26 | + | - | - | - | - | 0 |
|  |  | 27 | + | - | - | - | - | 0 |
|  |  | 28 | + | - | - | - | - | 0 |
|  |  | 29 | + | + | + | - | - | 0 |
|  |  | 30 | + | + | + | - | - | 0 |
|  |  | 31 | - | - | - | - | - | 0 |
|  |  | 32 | + | - | - | - | - | 0 |
|  |  | 33 | + | - | - | - | - | 0 |
|  |  | 34 | - | - | - | - | - | 0 |
|  |  | 35 | + | - | - | - | - | 0 |
|  |  | 36 | + | - | - | - | - | 0 |
|  |  | 37 | - | - | - | - | - | 0 |
| ∑ |  | 37 | 81.1% | 5.4% | 5.4% | 0% | 0% | 0% |
|  |  |  |  |  |  |  |  |  |

| Genotype of seed-lings, resistance | Comment | No. | Bracts/leaf structures & Flower structures^1^ counted together | | | | | Seeds/Comments |
| --- | --- | --- | --- | --- | --- | --- | --- | --- |
| *enp pid/enp pid (laterne) & ENP-P46T-GFP6, Hygrom.^res^* | Transgenic plant 1 descendants |  |  |  |  |  |  |  |
|  |  | 1-85 | 21 | | | | | 0 in all *laterne* plants |
| ∑ |  | 85 | 24.7% | | | | | 0% |
|  |  |  |  |  |  |  |  |  |
| Plant genotype | Comment | No. | Bracts/leaf structures & Flower structures^1^ counted together | | | | | Seeds/Comments |
| *enp pid/enp pid (laterne) & ENP-Y409E-GFP6, Hygrom.^res^* | Transgenic plant 1/2 descendants combined |  |  |  |  |  |  |  |
|  |  | 1-23 | 0 | | | | | 0 in all *laterne* plants |
| ∑ |  | 23 | 0% | | | | | 0% |
|  |  |  |  |  |  |  |  |  |
| Plant genotype | Comment | No. | Bracts/leaf structures & Flower structures^1^ counted together | | | | | Seeds/Comments |
| *enp pid/enp pid (laterne) & ENP-Y409A-GFP6, Hygrom.^res^* | Transgenic plant 1/2 descendants combined |  |  |  |  |  |  |  |
|  |  | 1-29 | 3 | | | | | 0 in all *laterne* plants |
| ∑ |  | 29 | 10.3% | | | | | 0% |
|  |  |  |  |  |  |  |  |  |

| Genotype of seed-lings, resistance | Comment | No. | Bracts/leaf structures & Flower structures^1^ counted together | | | | | Seeds/Comments |
| --- | --- | --- | --- | --- | --- | --- | --- | --- |
| *enp pid/enp pid (laterne) & MEL4 full length = EYFP-MEL4, Basta^res.^* | Transgenic plant 1 descendants |  |  |  |  |  |  |  |
|  |  | 1-63 | 0 | | | | | 0 in all *laterne* plants |
| ∑ |  | 63 | 0% | | | | | 0% |
|  |  |  |  |  |  |  |  |  |
| Genotype of seed-lings, resistance | Comment | No. | Bracts/leaf structures & Flower structures^1^ counted together | | | | | Seeds/Comments |
| *enp pid/enp pid (laterne) & MEL4-ENPCterm_long-GFP6, Hygrom.^res^* | Transgenic plant 1 descendants |  |  |  |  |  |  |  |
|  |  | 1-14 | 0 | | | | | 0 in all *laterne* plants |
| ∑ |  | 14 | 0% | | | | | 0% |
|  |  |  |  |  |  |  |  |  |
| Genotype of seed-lings, resistance | Comment | No. | Bracts/leaf structures & Flower structures^1^ counted together | | | | | Seeds/Comments |
| *enp pid/enp pid (laterne) & MEL4-ENPCterm_short-GFP6, Hygrom.^res^* | Transgenic plant 1 descendants |  |  |  |  |  |  |  |
|  |  | 1-34 | 0 | | | | | 0 in all *laterne* plants |
| ∑ |  | 34 | 0% | | | | | 0% |
|  |  |  |  |  |  |  |  |  |
